# Association between Body Composition and Bone Mineral Density in Children and Adolescents: A Systematic Review and Meta-Analysis

**DOI:** 10.3390/ijerph182212126

**Published:** 2021-11-18

**Authors:** Kai-Li Deng, Wan-Yu Yang, Jin-Li Hou, Hui Li, Hao Feng, Su-Mei Xiao

**Affiliations:** 1 Department of Epidemiology, School of Public Health, Sun Yat-sen University, Guangzhou 510080, China; dengkli@mail2.sysu.edu.cn (K.-L.D.); yangwy29@mail2.sysu.edu.cn (W.-Y.Y.); houjli@mail2.sysu.edu.cn (J.-L.H.); lihui256@mail2.sysu.edu.cn (H.L.); fenghao_chn@163.com (H.F.); 2 Guangdong Provincial Key Laboratory of Food, Nutrition and Health, School of Public Health, Sun Yat-sen University, Guangzhou 510080, China

**Keywords:** lean mass, fat mass, body fat percentage, bone mineral density, pediatric population, children, adolescent

## Abstract

Background: Bone mass acquisition during growth is a major determinant of the risk of developing osteoporosis later in life. Body composition is an anthropometric determinant of bone mineral density (BMD) and significantly influences its development during childhood and adolescence. Objective: This study aimed to systematically examine the association between body composition and bone mineral density in children and adolescents. Methods: Observational studies addressing this association were identified from PubMed (MEDLINE), Embase, Scopus and the Cochrane Library (up to January 2021). The study populations consisted of healthy children and adolescents. The DerSimonian and Laird method was used to compute pooled estimates of effect size and the respective 95% confidence intervals for upper limbs, femoral neck (FN), lumbar spine (LS) and total body, respectively. Subgroup analyses were further performed based on age, sex and ethnicity. Results: Thirty-one published studies were eligible for inclusion in this systematic review and meta-analysis, including three longitudinal studies. The combined population from all the studies amounted to 21,393 (11,205 males and 10,188 females). The pooled estimates of the correlation coefficients for lean mass (LM) and BMD ranged from 0.53 to 0.74 (*p* < 0.050), and the pooled regression coefficients ranged from 0.23 to 0.79 for FN, LS and total body (*p* < 0.050). For fat mass (FM), the pooled correlation coefficients ranged from 0.10 to 0.50 (*p* < 0.050) and the pooled regression coefficient was only significant for FN BMD with a weak strength (pooled β = 0.07, *p* < 0.050). The pooled regression coefficients for body fat percentage (BF%) were between −0.54 and −0.04 (*p* < 0.050). The subgroup analysis revealed a stronger association in Asians than in Caucasians for LM and in males compared to females for BF% (*p* < 0.050). Conclusions: This systematic review and meta-analysis supports a positive association between LM and BMD. BF% appears to have a deleterious effect on bone acquisition in children and adolescents.

## 1. Introduction

Osteoporosis is an important public health problem affecting millions of people worldwide [1]. It is characterized by low bone mass and microarchitecture disruption, leading to high risk of fracture. Low peak bone mass is a major risk factor for the development of osteoporosis in later life. Peak bone mass is defined as the amount of bone mass accumulated after attaining skeletal maturity [2]. It is well-known that childhood and adolescence are periods of significant skeletal development and maturation. Bone mass increases by approximately 45% during puberty. By the end of puberty, the bone mass reaches close to 90% of the adult peak bone mass [3]. It is essential to improve and maximize peak bone mass during childhood and adolescence to reduce the risk of developing osteoporosis later in life [4].

Body composition, i.e., lean mass (LM) and fat mass (FM), are reported to be closely associated with bone mineral density (BMD) in children and adolescents. LM is unambiguously associated with increases in BMD owing to the mechanical load added to the skeleton. The relationship between FM and bone mass, however, remains unclear. FM was initially considered to be a protective factor for bone health due to the increase in loading that occurs as a result of a higher total body mass; this effect is thought to be meditated by various osteokines, adipokines and myokines [5]. However, recent reports have indicated a complex relationship between FM and bone health. Adipose tissue may have unfavorable effects on skeletal health owing to inflammation, oxidative stress and the derivation of both adipocytes and osteoblasts from mesenchymal stem cell progenitors [6]. A meta-analysis of the influence of adipose tissue on bone mass in adults found that FM is positively correlated with BMD and the FM percentage is related to low bone mass [7].

To date, no quantitative systematic review has been published on the associations of LM, FM and body fat percentage (BF%) with bone parameters in children and adolescents, even though dozens of related investigations have been conducted. In 2016, Sioen et al. performed a qualitative systematic review on the relationship between body composition and BMD in children and adolescents [8]. Aside from reporting consistently positive associations between LM and BMD, the review was unable to clearly infer the relationship between absolute or relative FM and BMD. Since the publication of this systematic review, more than 10 studies on body composition and bone health in children and adolescents have been published [9,10,11,12,13,14,15,16,17,18,19,20,21]. In addition, there is a lack of studies that systematically analyze the skeletal site-, sex-, age- and ethnicity-specific associations between body composition and bone health in children and adolescents. Therefore, the current systematic review and meta-analysis aimed to analyze the associations of body composition, i.e., LM, FM and BF%, with BMD in the upper limbs, femoral neck (FN), lumber spine (LS) and total body, respectively, in children and adolescents, and to further examine whether the relationship differs based on sex, age and ethnicity.

## 2. Materials and Methods

The protocol adopted in this systematic review and meta-analysis was based on the Meta-Analysis of Observational Studies in Epidemiology (MOOSE) statements [22] and the Reporting Items for Systematic Reviews and Meta-Analyses (PRISMA) frameworks [23]. This systematic review and meta-analysis has been registered in the International Prospective Register of Systematic Reviews (PROSPERO; http://www.crd.york.ac.uk/prospero; accessed on 2 September 2021) database under the registration number CRD42021232700.

### 2.1. Search Strategy

Searches of the literature were conducted in PubMed (MEDLINE), Web of Science, Embase, Scopus and the Cochrane Library from the time of inception of each database up to January 2021. We restricted the search species to *Homo sapiens* and an age range between 0 and 19 years. The following search terms were used: (adolescent* OR teen* OR child* OR student*) AND (“bone density” OR “bone mineral density” OR “bone mass” OR “bone health” OR “bone phenotype” OR “bone parameter” OR “bone geometry” OR “BMD”) AND (“body composition” OR “lean mass” OR “fat free mass” OR “fat mass” OR “body fat percentage” OR “LM” OR “FFM” OR “FM”). The reference lists of the articles included and of previous relevant systematic reviews and meta-analyses were reviewed to obtain additional relevant studies.

### 2.2. Selection Criteria

Original research articles on the relationship between body composition and bone outcomes in children and adolescents were included in this systematic review and meta-analysis. The following inclusion criteria were used: (i) published in peer-reviewed journals; (ii) study participants aged 0–19 years; (iii) data obtained from observational studies; and (iv) body composition and bone parameters assessed by dual-energy X-ray absorptiometry (DXA) and/or quantitative computed tomography (QCT).

The exclusion criteria were as follows: (i) studies published twice or multiple times; (ii) no access to full text; (iii) studies not written in English; (iv) study participants with diseases other than non-pathological obesity; (v) comments, reviews or editorials; (vi) incomplete data; (vii) LM, FM and/or BF% not included; (viii) BMD not included; and (ix) presence of interventions. The literature search was implemented independently by two reviewers, and any inconsistencies were resolved by consensus after a detailed discussion with a third reviewer.

### 2.3. Data Extraction and Quality Assessment

The following information was extracted from the selected studies: (1) author name; (2) country or region of study; (3) year of publication; (4) study design; (5) characteristics of participants, i.e., sex, age and ethnicity; (6) sample size; (7) details of body composition, i.e., LM, FM and BF%; (8) skeletal site of BMD, i.e., upper limbs, LS, FN and total body; (9) types of measurement tools used to determine body composition and BMD; (10) confounding factors; and (11) correlation and/or regression coefficients.

The quality assessment tool for observational cohort and cross-sectional studies from the National Institutes of Health (NIH; https://www.nhlbi.nih.gov/health-topics/study-quality-assessment-tools, accessed on 15 November 2021) was used to evaluate the risk of bias in the included studies. The tool has 14 components: (a) research question; (b) population definition; (c) participation rate; (d) recruitment; (e) sample size; (f) analysis; (g) time frame; (h) exposure levels; (i) exposure measures; (j) exposure assessment; (k) outcome measures; (l) blinding; (m) loss to follow-up; and (*n*) confounding variables. Using these criteria, each study was rated as either good (i.e., most criteria met and a low risk of bias), fair (i.e., some criteria met and a moderate risk of bias) or poor (i.e., few criteria met and a high risk of bias). Both the data extraction and quality assessment of the included studies were performed by two independent reviewers, and any inconsistencies were settled by discussion with a third investigator.

### 2.4. Data Synthesis and Analysis

The analyzed variables were the correlation coefficients and regression coefficients between body composition and BMD. The three body composition measures were LM, FM and BF%. Four BMD sites were studied, including the upper limb, LS, FN and total body. Thus, a maximum of 12 correlation coefficients and 12 regression coefficients were extracted and analyzed. In the meta-analysis, the correlation coefficients were directly synthesized. For the regression coefficients, if the standardized regression coefficient was not provided, then the raw regression coefficient and standard deviation were used to estimate the standardized values. Heterogeneity among the studies was assessed using the standard *Q* test and *I*^2^ statistics. Random-effects estimates and the DerSimonian and Laird method were used in this study. The results were graphically displayed as forest plots. On this basis, subgroup analyses by gender, age and ethnicity were also carried out as specified in the analysis protocol. If a subgroup had fewer than two independent original studies, the meta-analysis was not conducted and the data were not displayed for this subgroup. Sensitivity analyses were conducted to assess the robustness of the summary estimates. The impacts of each result on the overall findings were examined by deleting each study from the model once. The difference between the two estimated effect sizes (ESs) was evaluated. Publication bias was evaluated using Egger’s test. Duval and Tweedie’s trim and fill test was used to correct for the effect of other possible biases. All analyses were performed using the STATA software package (vision 11.0). A two-tailed *p* < 0.050 was considered to indicate statistical significance.

## 3. Results

### 3.1. Characteristics of Eligible Studies

**Figure 1:**
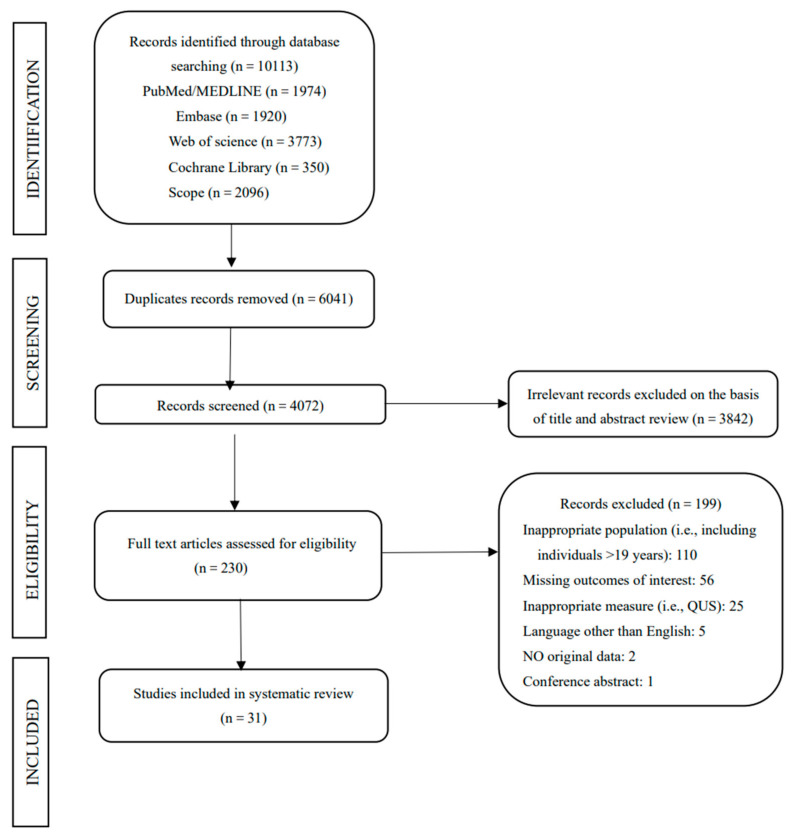
PRISMA flow diagram.

The flow diagram of the literature search process is shown in Figure 1. After excluding duplicates, the remaining 4072 articles were screened for titles and abstracts. Two hundred and thirty articles went through the full-text review after exclusion of articles that were either not population-based epidemiological studies or not relevant to the research question, i.e., did not investigate the association between body composition and bone mass. Finally, 31 studies were included in this systematic review and meta-analysis. The included publications consisted of 28 cross-sectional studies and 3 longitudinal studies [15,24,25]. Eleven studies were conducted in Europe [13,17,18,24,25,26,27,28,29,30,31], nine in Asia [11,12,15,20,21,32,33,34,35], seven in North America [10,14,16,19,36,37,38], three in South America [9,39,40] and one in Oceania [41]. Twenty-eight studies used DXA machines to assess bone parameters and body composition, and three studies applied peripheral QCT for the measurement. Fourteen studies [13,14,16,17,18,19,29] only reported the value of the regression coefficient, nine studies [9,11,20,30,31,32,39,40,41] only reported the value of the correlation coefficient and eight studies [25,26,27,28,33,36,37,38] reported both. Two studies investigated the association between LM and upper limb BMD, ten investigated the association between LM and LS BMD, eight investigated the association between LM and FN BMD and thirteen investigated the association between LM and total body BMD. Three studies investigated the association between FM and upper limb BMD, eight investigated the association between FM and LS BMD, nine investigated the association between FM and FN BMD and thirteen investigated the association between FM and total body BMD. Three studies investigated the association between BF% and LS BMD, three investigated the association between BF% and FN BMD and four investigated the association between BF% and total body BMD.

The majority (*n* = 13) of the reported correlation coefficients were not adjusted for confounding factors, but all of the reported regression coefficients were adjusted for multiple confounding factors such as age, height, weight or sex. Data were available on 21,393 participants (11,205 boys and 10,188 girls). The participants in these studies were healthy children and adolescents aged 4.8 to 19.0 years. Detailed characteristics of each study included are presented in Table 1.

### 3.2. Risk of Bias

The risk of bias was evaluated using the quality assessment tool for observational cohort and cross-sectional studies from the National Institutes of Health (NIH). Each included study was evaluated on 14 assessment items. We found that 83.9% (*n* = 26) of the studies had a moderate risk of bias and 16.1% (*n* = 5) had a low risk of bias. Items such as sample size, participation rate, exposure definition, outcome measurement and confounding bias were carefully evaluated. For cross-sectional studies, when analyzed by individual domain, the main shortcoming was in the field of time frame. The details of the quality assessments are listed in Supplementary Table S1.

### 3.3. Meta-Analysis

**Figure 2:**
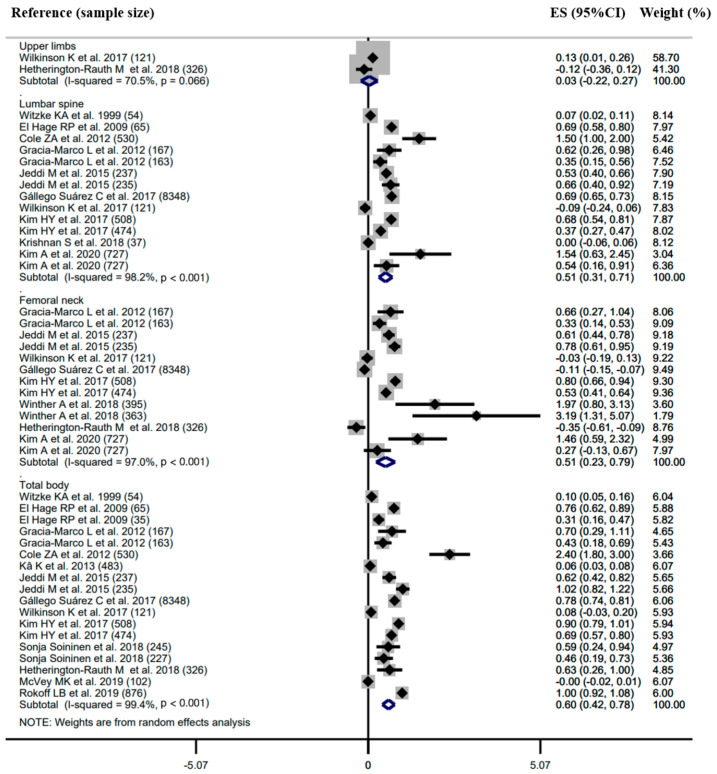
Forest plot of association (regression coefficient) between lean mass (LM) and bone mineral density (BMD) in upper limbs, lumber spine, femoral neck and total body, respectively. The effect size (ES) and 95% confidence interval (CI) for fully adjusted random effects are depicted for each study.

**Figure 3:**
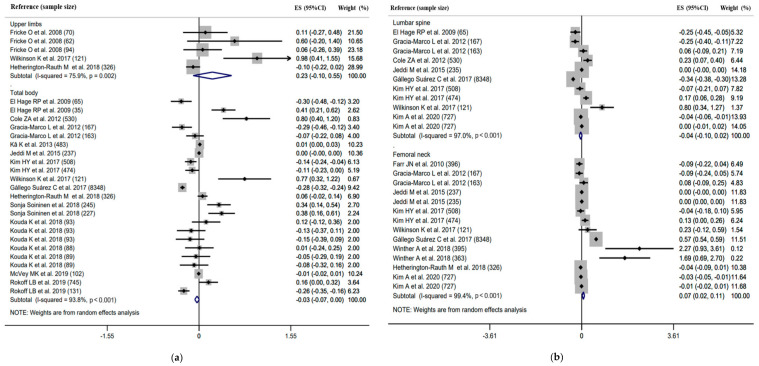
Forest plot of association (regression coefficient) between fat mass (FM) and bone mineral density (BMD) in (**a**) upper limbs, total body, (**b**) lumber spine, femoral neck, respectively. The effect size (ES) and 95% confidence interval (CI) for fully adjusted random effects are depicted for each study.

**Figure 4:**
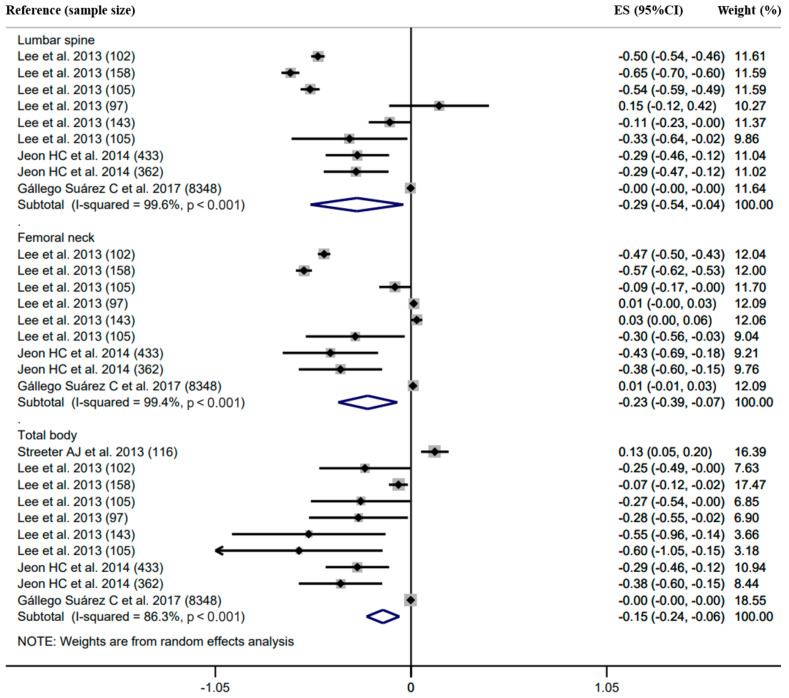
Forest plot of association (regression coefficient) between body fat percentage (BF%) and bone mineral density (BMD) in lumber spine, femoral neck and total body, respectively. The effect size (ES) and 95% confidence interval (CI) for fully adjusted random effects are depicted for each study.

Forest plots with the pooled correlation/regression estimates, corresponding 95% confidence interval (CI) and *I*^2^ heterogeneity statistics for body composition and BMD are shown in Figure 2, Figure 3 and Figure 4 and Supplementary Figures S1–S3.

#### 3.3.1. Correlation between Body Composition and BMD

A total of 17 studies reported the correlation coefficient between body composition (i.e., LM, FM and BF%) and BMD at the skeletal sites of the upper limb, FN, LS and total body (Supplementary Figures S1–S3).

There was a relatively high positive correlation between LM and BMD. The pooled correlation coefficients were 0.64 (95%CI 0.55–0.74, *I*^2^ = 82.9%, *n* = 6) for the FN BMD, 0.61 (95%CI 0.53–0.69, *I*^2^ = 78.7%, *n* = 9) for the LS BMD and 0.61 (95%CI 0.55–0.68, *I*^2^ = 96.5%, *n* = 17) for the total body BMD. FM had a moderately positive correlation with BMD. The pooled estimates for the correlation coefficient were 0.25 (95%CI 0.10–0.40, *I*^2^ = 88.3%, *n* = 7) for the FN BMD, 0.36 (95%CI 0.25–0.47, *I*^2^ = 79.0%, *n* = 9) for the LS BMD and 0.40 (95%CI 0.29–0.50, *I*^2^ = 94.3%, *n* = 17) for the total body BMD. No differences in the effect sizes of either the LM or FM for the different BMD skeletal sites were observed (*p* > 0.050).

BF% was negatively correlated with FN BMD (pooled *r* = −0.33, 95%CI −0.51 to −0.15, *I*^2^ = 89.5%, *n* = 4). It had no significant correlations with BMD at the other sites (pooled *r* = 0.03, 95%CI −0.31 to 0.38, *I*^2^ = 94.7%, *n* = 5 for upper limb; pooled *r* = −0.09, 95%CI −0.35 to 0.16, *I*^2^ = 95.5%, *n* = 7 for LS; pooled *r* = −0.04, 95%CI −0.22 to 0.15, *I*^2^ = 93.7%, *n* = 11 for total body). BMD at the different skeletal sites showed significantly different effect sizes for BF% (*p* < 0.050).

##### Subgroup Analysis

As shown in Supplementary Table S2, the subgroup analysis based on ethnicity showed that both LM and FM had stronger positive correlations with BMD in Asians than in Caucasians (*p* < 0.050). For LM, the pooled correlation coefficients were 0.54 (95%CI 0.24–0.83, *I*^2^ = 93.2%, *n* = 2) for the LS BMD, 0.75 (95%CI 0.71–0.79, *I*^2^ = 58.1%, *n* = 2) for the FN BMD and 0.77 (95%CI 0.70–0.84, *I*^2^ = 96.3%, *n* = 5) for the total body BMD in Asians, and 0.52 (95%CI 0.42–0.62, *I*^2^ = 65.6%, *n* = 5) for the LS BMD, 0.51 (95%CI 0.43–0.59, *I*^2^ = 0.0%, *n* = 3) for the FN BMD and 0.46 (95%CI 0.29–0.63, *I*^2^ = 96.2%, *n* = 10) for the total body BMD in Caucasians. For FM, the pooled correlation coefficients were 0.48 (95%CI 0.39–0.57, *I*^2^ = 36.4%, *n* = 2) for the LS BMD, 0.31 (95%CI 0.23–0.39, *I*^2^ = 47.2%, *n* = 2) for the FN BMD and 0.53 (95%CI 0.38–0.67, *I*^2^ = 94.9%, *n* = 5) for the total body BMD in Asians, and 0.30 (95%CI 0.16–0.43, *I*^2^ = 44.5%, *n* = 5) for the LS BMD, 0.20 (95%CI 0.03–0.36, *I*^2^ = 78.4%, *n* = 4) for the FN BMD and 0.35 (95%CI 0.19–0.51, *I*^2^ = 93.7%, *n* = 10) for the total body BMD in Caucasians.

In Supplementary Table S3, the results show that the correlation coefficients between LM and BMD at most skeletal sites were higher in boys than in girls. The correlation coefficients were 0.66 (95%CI 0.55–0.76, *I*^2^ = 70.2%, *n* = 4) for the LS BMD, 0.58 (95%CI 0.38–0.78, *I*^2^ = 87.9%, *n* = 3) for the FN BMD and 0.55 (95%CI 0.36–0.75, *I*^2^ = 97.1%, *n* = 7) for the total body BMD in boys, and 0.48 (95%CI 0.25–0.71, *I*^2^ = 87.9%, *n* = 4) for the LS BMD, 0.67 (95%CI 0.59–0.75, *I*^2^ = 76.3%, *n* = 2) for the FN BMD and 0.52 (95%CI 0.32–0.72, *I*^2^ = 96.6%, *n* = 7) for the total body BMD in girls. In contrast, FM showed higher positive correlations with BMD in girls than in boys (Supplementary Table S3). The pooled correlation coefficients were 0.45 (95%CI 0.31–0.58, *I*^2^ = 71.3%, *n* = 4) for the LS BMD, 0.30 (95%CI 0.05–0.54, *I*^2^ = 93.1%, *n* = 3) for the FN BMD and 0.44 (95%CI 0.25–0.63, *I*^2^ = 93.2%, *n* = 7) for the total body BMD in girls, and 0.30 (95%CI 0.04–0.42, *I*^2^ = 76.1%, *n* = 4) for the LS BMD, 0.18 (95%CI 0.02–0.34, *I*^2^ = 58.9%, *n* = 3) for the FN BMD and 0.35 (95%CI 0.15–0.54, *I*^2^ = 94.4%, *n* = 7) for the total body BMD in boys. No significant differences were found in correlation coefficients between females and males (*p* > 0.050).

No significant differences were observed for the correlation coefficients of BF% and BMD between ethnicities, nor between genders (*p* > 0.050, Supplementary Tables S2 and S3).

As shown in Supplementary Table S4, the subgroup analysis based on age showed that both LM and FM had stronger positive correlations with total body BMD in adolescents than in children (*p* < 0.050). The pooled correlation coefficients were 0.57 (95%CI 0.45–0.69, *I*^2^ = 89.6%, *n* = 9) for LM and 0.43 (95%CI 0.27–0.58, *I*^2^ = 91.0%, *n* = 9) for FM in adolescents, and 0.30 (95%CI −0.12 to 0.73, *I*^2^ = 97.7%, *n* = 3) for LM and 0.14 (95%CI −0.16 to 0.44, *I*^2^ = 93.4%, *n* = 3) for FM in children.

##### Sensitivity Analysis

Sensitivity analyses were performed by removing one study at a time, following which a Galbraith diagram was produced. The results indicated that some studies, i.e., Jeddi et al. [33], Mosca et al. [40] and Witzke et al. [38], may have been sources of heterogeneity. Two of these studies reported results from populations that had almost ten-year age ranges [33,40]. In addition, Jeddi et al. [33] pooled the girls and boys together for the analysis without a gender-based subgroup analysis. Witzke et al. [38] conducted their investigation in the year 1999 with a small sample size (*n* = 54), and all the other studies included were performed after 2008. After eliminating these studies, the heterogeneity decreased significantly and the results remained consistent (Supplementary Figures S4–S6). The overall estimates for the correlation coefficients between body composition (i.e., LM, FM and BF%) and BMD (i.e., upper limb, FN, LS and total body) were not significantly modified in either magnitude or direction when single studies were individually excluded from the meta-analysis (Supplementary Figures S7–S9).

##### Publication Bias

No potential publication bias was revealed by Egger’s test (*p* > 0.050), except for LM, FM and total body BMD, and FM and LS BMD (*p* < 0.050, Supplementary Table S8). However, none of studies were trimmed and filled when Duval and Tweedie’s trim and fill test was applied for the detection of publication bias (Supplementary Figure S10). This indicates that other potential biases, such as a language bias or inflated estimates from flawed methodological design in smaller studies, may have been responsible for the observed asymmetry.

#### 3.3.2. Association between Body Composition and BMD

Twenty-two studies reported the regression coefficient between body composition (i.e., LM, FM and BF%) and BMD at the skeletal sites of the upper limbs, FN, LS and total body (Figure 2, Figure 3 and Figure 4).

There was a positive association between LM and BMD (Figure 2). The pooled estimates of the regression coefficient were 0.03 (95%CI −0.22 to 0.27, *I*^2^ = 70.5%, *n* = 2) for the upper limb BMD, 0.51 (95%CI 0.31 to 0.71, *I*^2^ = 98.2%, *n* = 14) for the LS BMD, 0.51 (95%CI 0.23 to 0.79, *I*^2^ = 97.0%, *n* = 13) for the FN BMD and 0.60 (95%CI 0.42 to 0.78, *I*^2^ = 99.4%, *n* = 18) for the total body BMD. No evidence of differences was obtained for the values of the pooled β estimated across different BMD sites, i.e., LS, FN and total body (*p* > 0.050).

FM showed a significantly positive association with FN BMD, but the strength was weak (pooled β = 0.07, 95%CI 0.02 to 0.11, *I*^2^ = 99.4%, *n* = 14; Figure 3). No significant association was observed for FM and BMD at the other skeletal sites (pooled β = 0.23, 95%CI −0.10 to 0.55, *I*^2^ = 75.9%, *n* = 5 for upper limbs; pooled β = −0.04, 95%CI −0.10 to 0.02, *I*^2^ = 97.0%, *n* = 11 for LS; pooled β = −0.03, 95%CI −0.07 to <0.01, *I*^2^ = 93.8%, *n* = 23 for total body). A difference in the effect size was observed between FN and the other skeletal sites in FM (*p* < 0.050).

The association between BF% and BMD was negative (Figure 4). The summary estimates of the regression coefficient were −0.29 (95%CI −0.54 to −0.04, *I*^2^ = 99.6%, *n* = 9) for the LS BMD, −0.23 (95%CI −0.39 to −0.07, *I*^2^ = 99.4%, *n* = 9) for the FN BMD and −0.15 (95%CI −0.24 to −0.06, *I*^2^ = 86.3%, *n* = 10) for the total body BMD. A comparison of the values of the pooled β estimated across the different BMD sites demonstrated homogeneity for BF% (*p* > 0.050).

##### Subgroup Analysis

A significantly stronger association between LM and BMD was observed in Asians compared to Caucasians (*p* < 0.050, Supplementary Table S5). The pooled regression coefficients were 0.57 (95%CI 0.42–0.73, *I*^2^ = 74.8%, *n* = 6) for the LS BMD, 0.66 (95%CI 0.50–0.81, *I*^2^ = 72.6%, *n* = 6) for the FN BMD and 0.80 (95%CI 0.64–0.97, *I*^2^ = 79.0%, *n* = 4) for the total body BMD in Asians, and 0.44 (95%CI 0.16–0.72, *I*^2^ = 99.0%, *n* = 8) for the LS BMD, 0.22 (95%CI −0.05 to 0.49, *I*^2^ = 90.2%, *n* = 7) for the FN BMD and 0.53 (95%CI 0.34–0.72, *I*^2^ = 99.4%, *n* = 14) for the total body BMD in Caucasians. 

No significant difference was found for the associations of LM and BMD between genders (*p* > 0.050, Supplementary Table S6). However, the pooled estimates of the regression coefficient were higher for males than for females. The values of the pooled β were 0.55 (95%CI 0.26–0.85, *I*^2^ = 94.2%, *n* = 6) for the LS BMD, 0.72 (95%CI 0.31–1.13, *I*^2^ = 93.3%, *n* = 6) for the FN BMD and 0.61 (95%CI 0.28–0.93, *I*^2^ = 95.5%, *n* = 6) for the total body BMD in boys, and 0.37 (95%CI 0.15–0.60, *I*^2^ = 92.6%, *n* = 4) for the LS BMD, 0.42 (95%CI 0.09–0.74, *I*^2^ = 91.6%, *n* = 6) for the FN BMD and 0.51 (95%CI 0.25–0.76, *I*^2^ = 95.3%, *n* = 7) for the total body BMD in girls.

As shown in Supplementary Table S6, a significant sexual difference was found for the association between BF% and BMD (*p* < 0.001). The pooled regression coefficients were −0.52 (95%CI −0.61 to −0.43, *I*^2^ = 90.6%, *n* = 4) for the LS BMD, −0.39 (95%CI −0.57 to −0.21, *I*^2^ = 97.0%, *n* = 4) for the FN BMD and −0.19 (95%CI −0.34 to −0.05, *I*^2^ = 67.3%, *n* = 4) for the total body BMD in boys, and −0.15 (95%CI −0.32 to 0.03, *I*^2^ = 67.2%, *n* = 4) for the LS BMD, −0.02 (95%CI −0.09 to 0.04, *I*^2^ = 83.6%, *n* = 4) for the FN BMD and −0.31 (95%CI −0.64 to 0.03, *I*^2^ = 90.0%, *n* = 5) for the total body BMD in girls.

No significant differences were observed for the regression coefficients for FM and BMD between ethnicities, nor between genders (*p* > 0.050, Supplementary Table S5 and S6).

As shown in Supplementary Table S7, the subgroup analysis based on age did not identify significant differences for the pooled regression coefficients of LM, FM and total body BMD between the adolescent and the child populations (*p* > 0.050). Similar to the results of the analyses in all the included studies, LM was positively associated with total body BMD and no significant association was detected between FM and total body BMD in either of the two subgroups.

##### Sensitivity Analysis

Sensitivity analyses were performed by eliminating one study at a time, following which a Galbraith diagram was produced. The results indicated that some studies, i.e., El Hage et al. [26], Gállego et al. [10] and Jeddi et al. [33], may have been sources of heterogeneity. Two of these studies [10,26] pooled both genders together for their analyses without adjustment of age and height, which are important confounding factors. Jeddi et al. [33] reported the results from a population with an almost ten-year age range. They also did not adjust height in the regression analysis. After removing these studies, the heterogeneity significantly decreased. All the results remained consistent (Supplementary Figures S11–S13), except for those for LM and FN BMD. The positive association became one of no significance (pooled β = −0.09, 95%CI −0.23 to 0.05, *I*^2^ = 60.1%). The overall estimates of regression coefficients between body composition (i.e., LM, FM and BF%) and BMD (i.e., in the upper limbs, FN, LS and total body) were not significantly modified in either magnitude or direction when single studies were individually excluded from the meta-analysis (Supplementary Figures S14–S16).

##### Publication Bias

Egger’s test revealed a potential publication bias for the associations of LM with the FN and total body BMD and of BF% with the LS and total body BMD (*p* < 0.050), but not for the others (*p* > 0.050, Supplementary Table S8). Duval and Tweedie’s trim and fill test was then applied to correct the results (Supplementary Figure S17). After the correction with possible missing studies (*n* = 0–10), the associations remained the same. This implies that the unpublished results did not affect the interpretation of the existing results.

## 4. Discussion

The accrual of bone mass during childhood and adolescence is a strong determinant of the risk of developing osteoporosis later in life [4]. This systematic review and meta-analysis analyzed the relationships of LM, FM and BF% with the BMD in the upper limbs, LS, FN and total body, respectively, using data from 31 studies that included 21,393 children and adolescents. We found that both LM and FM were positively correlated with BMD. In the meta-analysis of the regression coefficients, after adjusting for potential confounding factors, except for the weak association between FN and BMD, the positive correlations between FM and BMD at the other skeletal sites studied disappeared, but all associations remained robust for LM and BMD. BF% showed a negative association with BMD at all the skeletal sites. In addition, the subgroup analysis showed that the associations were stronger in Asians than in Caucasians for LM and in males than in females for BF%.

The BMD in the LS, FN and total body showed significant heterogeneity, with an *I*^2^ of 86.3% to 99.6%. The heterogeneity was mainly caused by, but not limited to, three studies [10,26,33]. Some of these studies reported the results from populations that had age ranges greater than ten years and/or included both genders, which might partly explain the heterogeneity. When these studies were excluded from the analyses, the heterogeneity dropped substantially and almost all of the original observations remained unperturbed. Another possible reason for heterogeneity could have been the introduction of different confounding factors into the multiple regression mode. For example, EI Hage et al. [26] did not adjust age and height in the analysis, which are two important confounding factors commonly adjusted in related studies. In addition, small sample sizes could also have been one of the sources of heterogeneity. Overall, the heterogeneity did not appear to substantially impact the results.

This study supports the positive association of LM with bone health in children and adolescents, which is in accordance with prior reports. Moreover, results from this study highlight the consistency of this association across different skeletal sites, such as the LS, FN and total body. Therefore, we were able to demonstrate a positive association of BMD with LM at both partial and whole sites. This relationship can be partially attributed to the mechanical loading added to the skeleton and greater body weight. Muscle mass is closely related to LM, which implies that strong muscle contraction and forceful osteogenic stimulation of the adjacent tissue can potentially determine the BMD [1,42]. Previous studies have shown that mechanical stimulation-related muscle glycogen metabolism and systemic changes can promote bone development [43,44]. These phenomena are captured by the term “functional muscle–bone unit”, which is used in mechanostat theory and describes the adaptation of bone to the maximum physiological load, i.e., the peak force exerted by muscles [8,45].

The subgroup analyses revealed that, given the consistency of the pooled correlation and regression coefficients, the contribution of LM to BMD might be more pronounced in Asians than in Caucasians. In agreement with this result, another systematic review and meta-analysis that included 20,226 adults aged 18–92 years also found that the correlation between LM and BMD was stronger in Asians than in Caucasians [46]. Based on the assumption that body fat is generally higher in the Caucasian pediatric population than in the Asian population [47], some studies on Caucasian teenagers have shown that FM is a strong indicator of BMD [13,27]. In contrast, most studies in Asians report that LM has a more significant impact on BMD [20,21,33,48]. According to a systematic review, the prevalence of overweight and obesity in children and adolescents reached 23.8% for boys and 22.6% for girls in 2013 in Caucasians while, at the same time, the prevalence was 12.9% for boys and 13.4% for girls in developing countries (i.e., Asian and South American countries) [49]. The body fat was also higher in Caucasians based on the data in our study. It can be tentatively assumed that, compared to Caucasians, LM has a more important role than FM for BMD in Asians across all age group. In addition, puberty is one of the most critical periods for differentiation of sex hormones’ concentration. Sex hormone concentration grows in mid-childhood [50]. Puberty, or sex hormone concentration, is a variable that has been suggested to have a significant impact on the relationship between body composition and bone mass [28,51]. In this study, the subgroup analysis showed that the pooled correlation coefficients of LM with total body BMD were higher in adolescents than in children. However, no significant difference in the pooled regression coefficients was observed between these two subgroups after the adjustment of confounding factors. This might be partly attributed to the limited number of studies. Few studies analyzed their relationship at different stages of puberty. Related investigations are warranted in the future.

In contrast to LM, the relationship between FM and bone parameters is less clear. The pooled correlation coefficients calculated in this study showed that FM was positively correlated with BMD. Moreover, except for the weak association between FN and BMD, the positive correlations between FM and BMD at the other three skeletal sites studied all disappeared. In the analyses of the correlation coefficients, the influence of the important confounding factors was not accounted for. For example, studies found that the FM contribution can be confounded by the LM or body weight. Gracia-Marco et al. [29], Cole et al. [24] and Hoy et al. [52] all found that the positive associations between FM and BMD were attenuated after adjusting for LM. In accordance with this, the summary estimates for the regression coefficients, in which the LM, weight or BMI were corrected accordingly, showed the same trends for the relationship between FM and BMD. Therefore, caution should be exercised when drawing conclusions from studies that report associations between FM and bone parameters without adjusting for LM or body weight. In addition, a previous study pointed out that the pattern of fat distribution may play a more important role than the total body fat in determining bone health [51]. Therefore, more emphasis should be given to the association between regional adiposity and bone health in the future.

Unlike the absolute adipose mass, relative adipose mass may be a more valuable predictor of BMD. The current study identified a significant negative relationship between BF% and BMD. A previous systematic review and meta-analysis of adults reported a parabolic element between the relative adipose mass and bone mass and a strong negative correlation during the bone growth (<25 years) and bone loss (>55 years) periods [7]. These findings suggest that the adverse impacts of high BF% are much stronger when bone metabolism is in a dynamic state than when it is maintained at a steady state. A number of mechanisms could explain this detrimental effect. For example, higher BF% is connected with increased oxidative stress, which negatively affects bone health. Reactive oxygen species mediate osteoclast differentiation and act as signaling molecules that regulate bone remodeling. However, under oxidative stress conditions, elevated levels of reactive oxygen species may cause a disproportionate increase in bone resorption, thereby increasing the rate of bone loss and leading to several bone disorders [53,54]. Also, obese individuals have been noted to have vitamin D deficiency. Vitamin D is an essential nutrient that plays an important role in calcium homeostasis [55]. In a paediatric population, an association between low vitamin D level and activation of pro-inflammatory pathways among the obese has been identified [56]. In addition, previous studies have reported a positive correlation between BF% and bone marrow adipose tissue (BMAT) [57,58]. BMAT is located within the confines of the skeleton, and its role in age-related osteoporosis has attracted significant attention in recent years. At the cellular level, both marrow adipocytes and osteoblasts originate from mesenchymal stem cells, and the competition implies that increased adipogenesis can lead to a decrease in osteogenesis [59]. Another hypothesis is that marrow adipocytes secrete inflammatory factors that affect osteoclast activity and therefore directly influence bone metabolism [60].

The subgroup analyses revealed that bone mass in boys was more susceptible to the negative influence of increased relative adipose mass compared to in girls. In accordance with this result, a previous systematic review and meta-analysis including 2587 participants also pointed out that BF% exerted a stronger deleterious effect on BMD in males than in females [7]. These results may be mainly explained by the influence of female hormones, such as estradiol, which is a critical and direct hormonal regulator of bone metabolism and present in greater concentrations in women than in men [61,62]. Adipose tissue, as an endocrine organ, is recognized as a significant site for transformation of sex steroid hormones and their action. Adipose tissue is also a key source of aromatase, which contributes to estrogen synthesis from androgen precursors [7,63]. These impacts related to estrogen could reduce the detrimental influence of adipose tissue on bone mass in girls to some extent. These data imply that more attention should be paid to the adverse effects of relative adiposity on bone mass in children and adolescents, especially in boys.

To the best of our knowledge, this is the first quantitative systematic review that addresses the relationship between body composition (i.e., LM, FM and BF%) and bone parameters (BMD in the upper limbs, LS, FN and total body) in children and adolescents. This systematic review and meta-analysis provides a comprehensive and thorough examination of the relationship between body composition and bone mass in children and adolescents. Nevertheless, the results must be interpreted in the context of certain limitations. First, due to practical reasons, some common systematic review and meta-analysis shortcomings were unavoidable (e.g., publication bias and limited accessibility of complete study information), although we conducted an extensive literature search and performed several checks by cross-referencing. Second, there is a consensus that the total body without the head and not the total body including the head should be used for such analyses, because the skull constitutes a large percentage of the skeleton and is not affected by environmental factors [1]. However, some of the included studies only had data for total body bone measurements that included the head. Third, it is known that age and puberty stage play an important role in bone development [51]. Due to the lack of specific data, this study could not examine the effects of age or puberty status on the relationships between body composition and BMD. Finally, most studies included in this study were cross-sectional and ranked low in the hierarchy of evidence provided by study designs. Given the nature of these studies, temporal ambiguity represents an insurmountable threat to cause–effect inferences. Therefore, large prospective cohort studies are required to achieve conclusive results in this field.

## 5. Conclusions

Body composition and bone mass are two important and closely related components of the human body. LM should be considered as a useful marker of bone mass during development and maturation. BF% appears to play a more prominent deleterious role than FM in bone acquisition. To optimize skeletal status and maximize peak bone mass acquisition, more emphasis should be placed on muscle-strengthening exercises and the reduction of fat tissue in children and adolescents.

## Figures and Tables

**Table 1:** Characteristics of the studies included in the systematic review and meta-analysis.

Author, Year	Country	Design	Sample Size (M, F)	Age (y)	Body Composition	Assessment	BMD Sites	Covariates	
						Body Composition/BMD		*r*	β
Witzke KA et al. 1999	USA	Cross-sectional study	54 (0, 54)	14.6 ± 0.50	LM, FM	DXA ^a^	WB, FN, LS (L2~L4)	None	LM: months past menarche, height, bone-free LM, leg strength, leg power, FM
Fricke O et al. 2008	Germany	Cross-sectional study	295 (139, 156)	Prepubertal M: 8.7 ± 1.90 F: 8.5 ± 1.60 Puberty M: 14.5 ± 2.40 F: 14.4 ± 2.80	LM, FM, BF%	pQCT ^d^	Arms	None	FM: sex, height, fat area, muscle area
Goulding et al. 2008	New Zealand	Cross-sectional study	194 (113, 81)	M: 5.0 ± 0.05, F: 5.0 ± 0.05	LM, FM	DXA ^b^	TBLH	LM/FM: sex, weight	NA
El Hage RP et al. 2009	France	Cross-sectional study	100 (65, 35)	M: 15.2 ± 0.60, F: 15.2 ± 0.70	LM, FM, BF%	DXA ^a^	WB, LS (L1~L4)	None	LM/FM: FM (or LM)
El Hage R et al. 2010	Lebanon	Cross-sectional study	65 (0, 65)	Obese: 15.5 ± 2.70 Overweight: 15.0 ± 1.80 Normal: 15.6 ± 1.60	LM, FM, BF%	DXA ^a^	WB	None	NA
Farr JN et al. 2010	USA	Cross-sectional study	396 (0, 396)	Tertiles of FM Lowest: 10.3 ± 1.00 Middle: 10.9 ± 1.10 Highest:11.0 ± 1.10	LM, FM, BF%	DXA ^b^/pQCT ^d^	FN	None	FM: muscle cross-sectional area, maturity, bone length, physical activity, ethnicity
Viljakainen HT et al. 2011	Finland	Cross-sectional study	186 (73, 113)	M: 11.7 (7.7–18.1) F: 13.2 (7.4–18.8)	BF%	DXA ^a^	WB, FN, LS (L1~L4)	BF%: age, pubertal development	NA
Cole ZA et al. 2012	England	Longitudinal study	499 (253, 246)	M: 6.6 ± 0.20 F: 6.6 ± 0.20	LM, FM	DXA ^a^	WB, LS	NA	FM: LM
Gracia-Marco L et al. 2012	Spain	Cross-sectional study	330 (167, 163)	M: 14.7 ± 1.30 F: 14.7 ± 1.10	LM, FM	DXA ^a^	WB, LS, FN	NA	LM/FM: height, calcium intake, sexual maturation, average physical activity, WB FM (or LM)
Júnior IF et al. 2013	Brazil	Cross-sectional study	175 (83, 92)	11.1 ± 2.60	BF%	DXA ^b^	WB	None	NA
Kâ K et al. 2013	Canada	Cross-sectional study	483 (305, 178)	9.4 ± 0.90	LM, FM	DXA ^b^	WB	LM/FM: age, height	LM/FM: age, sex, height, daily calcium, vitamin D intake, daily physical activity, vitamin and mineral intake in the two previous weeks, bone or joint problems including history of fracture, FM (or LM)
Lee K et al. 2013	Korea	Cross-sectional study	710 (365, 345)	10.0–19.0	BF%	DXA ^a^	Arms, WB, LS, FN	NA	BF%: age, weight, height, serum 25(OH) vitamin D level, calcium intake, menarche status
Ivuskans A et al. 2013	Estonia	Cross-sectional study	264 (264, 0)	Normal: 12.1 ± 0.77 Overweight: 11.9 ± 0.76	LM, FM, BF%	DXA ^b^	WB, FN, LS (L2~L4)	FM: age, pubertal status	NA
Streeter AJ et al. 2013	England	Prospective longitudinal study	347 (NA, NA)	Baseline M: 8.9 ± 0.02 F: 8.9 ± 0.02 Follow up 9, 10, 11, 12, 13, 14, 15, 16	BF%	DXA ^b^	TBLH	None	BF%: age at peak height velocity
Mosca LN et al. 2014	Brazil	Cross-sectional study	377 (170, 207)	10.0–19.0	LM, FM, BF%	DXA ^a^	WB, FN LS (L1~L4)	None	NA
Jeon HC et al. 2014	Korea	Cross-sectional study	795 (433, 362)	M: 15.2 ± 0.13 F: 15.0 ± 0.13	BF%	DXA ^a^	WB, LS, FN	NA	BF%: age, menarche status, height, weight, serum 25-OH vitamin D, physical activity, energy intake, calcium intake, LM of whole body
Jeddi M et al. 2015	Iran	Cross-sectional study	469 (235, 234)	9.0–18.0	LM, FM, BF%	DXA ^a^	WB, LS, FN	None	LM/FM: age, sex, stage of puberty, level of 25-hydroxy vitamin D, FM (or LM)
Ripka WL et al. 2016	Brazil	Cross-sectional study	318 (318, 0)	14.9 ± 1.52	LM, FM, BF%	DXA ^a^	Arms, WB, FN, LS	None	NA
Khwanchuea R et al. 2017	Thailand	Cross-sectional study	135 (0, 135)	16.1 ± 0.49	LM, FM, BF%	DXA ^c^	WB, LS (L2~L4)	None	NA
Kim HY et al. 2017	Korea	Cross-sectional study	982 (508, 474)	15.6 ± 0.10	LM, FM	DXA ^a^	FN, WB, LS (L1~L4)	NA	LM/FM: age, vitamin D deficiency, insufficient Ca intake, physically inactive, homeostasis model assessment of insulin resistance, FM (or LM)
Wilkinson K et al. 2017	England	Cross-sectional study	121 (121, 0)	13.1 ± 1.00	LM, FM	DXA ^b^	Arms, TBLH, FN, LS (L1~L4)	NA	LM/FM: height, age, physical activity, FM (or LM)
Gállego Suárez C et al. 2017	USA	Cross-sectional study	8348 (4745, 3603)	13.0 ± 4.40	LM, FM, BF%	DXA ^a^	WB, LS	NA	FM/BF%: gender, race, LM
Sonja Soininen et al. 2018	Finland	Cross-sectional study	472 (227, 245)	7.6 ± 0.40	LM, FM, BF%	DXA ^b^	TBLH	NA	LM/FM: age, sex, height, FM (or LM)
Krishnan S et al. 2018	USA	Cross-sectional study	37 (19, 18)	Overweight: 15.6 ± 2.12 Normal weight: 16.5 ± 2.59	LM	DXA ^a^	WB, LS	NA	LM: waist to hip ratio, truck to total fat ratio, percent trunk fat, C-reactive protein, total activity time, apo CIII ratio, gender, homeostatic model assessment-estimated insulin resistance
Kouda K et al. 2018	Japan	Longitudinal study	545 (279, 266)	Baseline M: 11.2 ± 0.30 F: 11.1 ± 0.30 Follow up 14.0	LM, FM	DXA ^a^	WB	NA	FM: pubic hair appearance, sedentary behavior, height
Winther A et al. 2018	Norway	Cross-sectional study	759 (364, 395)	M: 16.7 ± 0.40 F: 16.6 ± 0.40	LM, FM	DXA ^b^	FN	NA	LM/FM: age, height, sexual maturity, physical activity levels, calcium intake, vitamin D levels, alcohol consumption, smoking habits
Hetherington-Rauth M et al. 2018	USA	Cross-sectional study	326 (0, 326)	10.8 ± 1.10	LM, FM, BF%	DXA ^b^/pQCT ^d^	Arms, FN, WB	NA	LM/FM: maturity offset, height, ethnicity, FM (or LM)
McVey MK et al. 2019	Ireland	Cross-sectional study	102 (47, 55)	5.1 ± 0.13	LM, FM	DXA ^b^	WB	None	LM/FM: sex, maternal BMD, maternal education level, membership of intervention/control group, breastfeeding status
Rokoff LB et al. 2019	USA	Cross-sectional study	876 (430, 446)	7.7 ± 1.00	LM, FM	DXA ^a^	TBLH	NA	FM: maternal education, pubertal status, physical activity, environmental tobacco smoke exposure, 25(OH)D plasma concentration, maternal marital status, annual household income, sex, ethnicity, height, age, fat-free mass
Song C et al. 2019	China	Cross-sectional study	1179 (581, 598)	M: 11.8 ± 3.71 F: 12.4 ± 3.81	LM, FM	DXA ^a^	TBLH	None	NA
Kim A et al. 2020	Korea	Cross-sectional study	1454 (727, 727)	Total: 15.1 ± 0.60 M: 15.1 ± 0.08 F: 15.1 ± 0.09	LM, FM	DXA ^b^	LS, FN	NA	LM/FM: age, weight, walking, muscle-strengthening exercises, nutrition (intake of calcium and serum vitamin D)

Notes: R, correlation coefficient; β, regression coefficient; LM, lean mass; FM, fat mass; BF%, body fat percentage; LS, lumbar spine; FN, femoral neck; WB, whole body; TBLH, total body less head; M, male; F, female; NA, not available; a, Hologic (Discovery QDR, QDR 2000 plus, QDR 1000/w, QDR 4500 W, QDR Delphi series, Explorer scanner Bedford); b, GE-Lunar (DPX-NT, Madison WI); c, Perols; d, Stratec (XCT 3000, XCT 2000).

## Data Availability

Requests for data may be directed to the corresponding author and are subject to institutional data use agreements.

## Supplementary Materials

The following are available online at https://www.mdpi.com/article/10.3390/ijerph182212126/s1, Table S1: Study quality assessed by the Quality Assessment tool for Observational Cohort and Cross-Sectional Studies from the National Institutes of Health. Table S2: Subgroups analysis by ethnicity for correlation coefficients. Table S3: Subgroup analysis by sex for correlation coefficients. Table S4: Subgroups analysis by age for correlation coefficients. Table S5: Subgroups analysis by ethnicity for regression coefficients. Table S6: Subgroups analysis by sex for regression coefficients. Table S7: Subgroups analysis by age for regression coefficients. Table S8: Assessment of potential publication bias with Egger’s test. Figure S1: Forest plot of correlation coefficients between lean mass and bone mineral density in the lumber spine, femoral neck and total body, respectively. The effect size (ES) and 95% confidence interval (CI) for fully adjusted random effects are depicted for each study. Figure S2: Forest plot of correlation coefficients between fat mass and bone mineral density in the lumber spine, femoral neck and total body, respectively. The effect size (ES) and 95% confidence interval (CI) for fully adjusted random effects are depicted for each study. Figure S3: Forest plot of correlation coefficients between body fat percentage and bone mineral density in the upper limbs, lumber spine, femoral neck and total body, respectively. The effect size (ES) and 95% confidence interval (CI) for fully adjusted random effects are depicted for each study. Figure S4: Forest plot of the correlation coefficients between lean mass and bone mineral density in the lumbar spine (A), femoral neck (B) and total body (C) after the exclusion of studies with large heterogeneity. Figure S5: Forest plot of the correlation coefficients between fat mass and bone mineral density in the lumbar spine (A), femoral neck (B) and total body (C) after the exclusion of studies with large heterogeneity. Figure S6: Forest plot of the correlation coefficients between body fat percentage and bone mineral density in the lumbar spine (A), femoral neck (B) and total body (C) after the exclusion of studies with large heterogeneity. Figure S7: Plots of sensitivity analyses involving the removal of one study at a time. (A) Four studies reported the correlation coefficient between lean mass and femoral neck bone mineral density (BMD). (B) Six studies reported the correlation coefficient between lean mass and lumbar spine BMD. (C) Twelve studies reported the correlation coefficient between lean mass and total body BMD. Figure S8: Plots of sensitivity analyses involving the removal of one study at a time. (A) Five studies reported the correlation coefficient between fat mass and femoral neck BMD. (B) Six studies reported the correlation coefficient between fat mass and lumbar spine BMD. (C) Twelve studies reported the correlation coefficient between fat mass and total body BMD. Figure S9: Plots of sensitivity analyses involving the removal of one study at a time. (A) Two studies reported the correlation coefficient between body fat percentage and upper limb bone mineral density (BMD). (B) Three studies reported the correlation coefficient between body fat percentage and femoral neck BMD. (C) Five studies reported the correlation coefficient between body fat percentage and lumbar spine BMD. (D) Eight studies reported the correlation coefficient between body fat percentage and total body BMD. Figure S10: Plots of Duval and Tweedie’s trim and fill test. (A) No studies trimmed and filled for the correlation coefficient between lean mass and total body bone mineral density (BMD). (B) No studies trimmed and filled for the correlation coefficient between fat mass and lumbar spine BMD. (C) No studies trimmed and filled for the correlation coefficient between fat mass and total body BMD. Figure S11: Forest plot of the regression coefficient between lean mass and bone mineral density in the lumbar spine (A), femoral neck (B) and total body (C) after exclusion of a study that had large heterogeneity. Figure S12: Forest plot of the regression coefficient between fat mass and bone mineral density in the lumbar spine (A), femoral neck (B) and total body (C) after the exclusion of studies with large heterogeneity. Figure S13: Forest plot of the regression coefficient between body fat percentage and bone mineral density in the lumbar spine (A), femoral neck (B) and total body (C) after the exclusion of studies with large heterogeneity. Figure S14: Plots of sensitivity analyses involving the removal of one study at a time. (A) Two studies reported the regression coefficient between lean mass and upper limb bone mineral density (BMD). (B) Ten studies reported the regression coefficient between lean mass and lumbar spine BMD. (C) Eight studies reported the regression coefficient between lean mass and femoral neck BMD. (D) Thirteen studies reported the regression coefficient between lean mass and total body BMD. Figure S15: Plots of sensitivity analyses involving the removal of one study at a time. (A) Three studies reported the regression coefficient between fat mass and upper limb bone mineral density (BMD). (B) Eight studies reported the regression coefficient between fat mass and lumbar spine BMD. (C) Nine studies reported the regression coefficient between fat mass and femoral neck BMD. (D) Thirteen studies reported the regression coefficient between fat mass and total body BMD. Figure S16: Plots of sensitivity analyses involving the removal of one study at a time. (A) Three studies reported the regression coefficient between body fat percentage and lumbar spine bone mineral density (BMD). (B) Three studies reported the regression coefficient between body fat percentage and femoral neck BMD. (C) Four studies reported the regression coefficient between body fat percentage and total body BMD. Figure S17: Plots of Duval and Tweedie’s trim and fill test. (A) Six items were trimmed and filled for the regression coefficient between lean mass and femoral neck bone mineral density (BMD). (B) Ten items were trimmed and filled for the regression coefficient between lean mass and total body BMD. (C) No studies were trimmed and filled for the regression coefficient between body fat percentage and lumbar spine BMD. (D) No studies were trimmed and filled for the regression coefficient between body fat percentage and total body BMD.
