# Supplementary material for: Association between Body Composition and Bone Mineral Density in Children and Adolescents: A Systematic Review and Meta-Analysis
Source: Int J Environ Res Public Health. 2021 Nov 18;18(22):12126. doi: 10.3390/ijerph182212126 (PMC8618958; doi:10.3390/ijerph182212126)
Supplement: Supplementary file 1 [file ijerph-18-12126-s001.zip › ijerph-1468802-SI.pdf]

Association between body composition and bone parameter in children and adolescents: a systematic review and meta-analysis

Kai-Li Deng [1], Wan-Yu Yang [1], Jin-Li Hou [1], Hui-Li [1], Hao Feng[1] and Su-Mei Xiao [1,2,*]

[1] Department of Epidemiology, School of Public Health, Sun Yat-sen University, Guangzhou, Guangdong, China; dengkli@mail2.sysu.edu.cn
[2] Guangdong Provincial Key Laboratory of Food, Nutrition and Health, School of Public Health, Sun Yat-sen University; xiaosm3@mail.sysu.edu.cn
* Correspondence: xiaosm3@mail.sysu.edu.cn; Tel.: +86-20-8733-0151

Supplementary Table 1. Study quality assessed by The Quality Assessment tool for Observational Cohort and Cross-sectional Studies from the National Institutes of Health

	Author, year	Research question	Population	Participation rate	Recruitment Inclusion/Exclusion	Sample size	Analysis	Timeframe	Exposure levels	Exposure measures	Exposure assessment	Outcome measures	Outcome blinding	Loss of follow-up	Confounding variables	Risk of bias
1	Witzke KA et al. 1999	Y	Y	NA	Y	N	N	N	Y	Y	NA	Y	CD	NA	Y	Fair
2	Fricke O et al. 2008	Y	Y	NA	Y	N	N	N	Y	Y	NA	Y	CD	NA	N	Fair
3	Goulding et al. 2008	Y	Y	NA	Y	N	N	N	Y	Y	NA	Y	CD	NA	Y	Fair
4	El Hage RP et al. 2009	Y	Y	NA	Y	N	N	N	Y	Y	NA	Y	CD	NA	Y	Fair
5	El Hage R et al. 2010	Y	Y	NA	Y	N	N	N	Y	Y	NA	Y	CD	NA	Y	Fair
6	Farr JN et al. 2010	Y	Y	NA	Y	N	N	N	Y	Y	NA	Y	CD	NA	Y	Fair
7	Viljakainen HT et al. 2011	Y	Y	NA	N	N	N	N	Y	Y	NA	Y	CD	NA	Y	Fair

Notes: NA, not applicable; CD, cannot determine.

Supplementary Table 1. Continued

	Author, year	Research question	Population	Participation rate	Recruitment Inclusion/Exclusion	Sample size	Analysis	Timeframe	Exposure levels	Exposure measures	Exposure assessment	Outcome measures	Outcome blinding	Loss of follow-up	Confounding variables	Risk of bias
8	Cole ZA et al. 2012	Y	Y	N	Y	N	N	N	Y	Y	NA	Y	CD	Y	Y	Fair
9	Gracia-Marco L et al. 2012	Y	Y	NA	Y	N	N	N	Y	Y	NA	Y	CD	NA	Y	Fair
10	Júnior IF et al. 2013	Y	Y	NA	Y	Y	N	N	Y	Y	NA	Y	CD	NA	Y	Good
11	Kâ K et al. 2013	Y	Y	NA	Y	N	N	N	Y	Y	NA	Y	CD	NA	Y	Fair
12	Lee K et al. 2013	Y	Y	NA	N	N	N	N	Y	Y	NA	Y	CD	NA	Y	Fair
13	Ivuskans A et al. 2013	Y	Y	NA	Y	N	N	N	Y	Y	NA	Y	CD	NA	N	Fair
14	Streeter AJ et al. 2013	Y	Y	Y	Y	N	Y	Y	Y	Y	NA	Y	CD	N	Y	Good

Notes: NA, not applicable; CD, cannot determine.

Supplementary Table 1. Continued

	Author, year	Research question	Population	Participation rate	Recruitment Inclusion/Exclusion	Sample size	Analysis	Timeframe	Exposure levels	Exposure measures	Exposure assessment	Outcome measures	Outcome blinding	Loss of follow-up	Confounding variables	Risk of bias
15	Mosca LN et al. 2014	Y	Y	NA	Y	N	N	N	Y	Y	NA	Y	CD	NA	Y	Fair
16	Jeon HC et al. 2014	Y	Y	NA	Y	N	N	N	Y	Y	NA	Y	CD	NA	Y	Fair
17	Jeddi M et al. 2015	Y	Y	NA	Y	N	N	N	Y	Y	NA	Y	CD	NA	Y	Fair
18	Ripka WL et al. 2016	Y	Y	NA	Y	N	N	N	Y	Y	NA	Y	CD	NA	N	Fair
19	Khwanchuea R et al. 2017	Y	Y	NA	Y	N	N	N	Y	Y	NA	Y	CD	NA	N	Fair
20	Wilkinson K et al. 2017	Y	Y	NA	Y	N	N	N	Y	Y	NA	Y	CD	NA	Y	Good
21	Kim HY et al. 2017	Y	Y	NA	Y	N	N	N	Y	Y	NA	Y	CD	NA	Y	Fair
22	Gállego Suárez C et al.2017	Y	Y	NA	Y	N	N	N	Y	Y	NA	Y	CD	NA	Y	Fair

Notes: NA, not applicable; CD, cannot determine.

Supplementary Table 1. Continued

	Author, year	Research question	Population	Participation rate	Recruitment Inclusion/Exclusion	Sample size	Analysis	Timeframe	Exposure levels	Exposure measures	Exposure assessment	Outcome measures	Outcome blinding	Loss of follow-up	Confounding variables	Risk of bias
23	Sonja Soininen et al. 2018	Y	Y	NA	Y	N	N	N	Y	Y	NA	Y	CD	NA	Y	Fair
24	Krishnan S et al. 2018	Y	Y	NA	Y	N	N	N	Y	Y	NA	Y	CD	NA	Y	Fair
25	Kouda K et al. 2018	Y	Y	Y	Y	N	Y	Y	Y	Y	NA/Y	Y	CD	Y	Y	Good
26	Winther A et al. 2018	Y	Y	Y	Y	N	N	N	Y	Y	NA	Y	CD	NA	Y	Good
27	Hetherington M et al. 2018	Y	Y	NA	Y	N	N	N	Y	Y	NA	Y	CD	NA	Y	Fair
28	McVey MK et al. 2019	Y	Y	NA	Y	N	Y	Y	Y	Y	NA	Y	CD	NA	Y	Fair
29	Rokoff LB et al. 2019	Y	Y	NA	Y	N	N	N	Y	Y	NA	Y	CD	NA	Y	Fair
30	Song C et al. 2019	Y	Y	NA	Y	N	N	N	Y	Y	NA	Y	CD	NA	Y	Fair
31	Kim A et al. 2020	Y	Y	NA	Y	N	N	N	Y	Y	NA	Y	CD	NA	Y	Fair

Notes: NA, not applicable; CD, cannot determine.

Supplementary Table 2. Subgroups analysis by ethnicity for correlation coefficients

	White				Yellow				Brown			
	**n**	**ES (95%CI)**	***p***	***I*²**	**n**	**ES (95%CI)**	***p***	***I*²**	**n**	**ES (95%CI)**	***p***	***I*²**
**LM**												
Lumbar spine BMD	5	0.52(0.42, 0.62)	<0.001	65.6	2	0.54(0.24, 0.83)	<0.001	93.2	2	0.75(0.69, 0.81)	<0.001	0.0
Femoral neck BMD	3	0.51(0.43, 0.59)	<0.001	0.0	2	0.75(0.71, 0.79)	<0.001	58.1	2	0.72(0.66, 0.79)	<0.001	2.6
Total body BMD	10	0.46(0.29, 0.63)	<0.001	96.2	5	0.77(0.70, 0.84)	<0.001	96.3	2	0.72(0.65, 0.79)	<0.001	0.0
**FM**												
Lumbar spine BMD	5	0.30(0.16, 0.43)	<0.001	44.5	2	0.48(0.39, 0.57)	<0.001	36.4	2	0.34(-0.15, 0.83)	0.170	93.9
Femoral neck BMD	4	0.20(0.03, 0.36)	0.002	78.4	2	0.31(0.23, 0.39)	<0.001	47.2	2	0.29(-0.22, 0.79)	0.264	94.0
Total body BMD	10	0.35(0.19, 0.51)	<0.001	93.7	5	0.53(0.38, 0.67)	<0.001	94.9	2	0.19(-0.42, 0.80)	0.543	95.9
**BF%**												
Lumbar spine BMD	2	<0.01(-0.42, 0.42)	0.996	78.1	2	0.25(0.09, 0.40)	0.002	69.4	4	-0.38(-0.45, -0.31)	<0.001	<0.1
Total body BMD	2	-0.06(-0.24, 0.12)	0.530	66.7	2	0.24(-0.18, 0.66)	0.260	96.3	3	-0.17(-0.48, 0.14)	0.274	95.2

Notes: LM, lean mass; FM, fat mass; BF%, body fat percentage; ES, effect size; CI, confidence interval; BMD, bone mineral density.

Supplementary Table 3. Subgroup analysis by sex for correlation coefficients

	Female				Male			
	**n**	**ES (95%CI)**	***p***	***I^2***	**n**	**ES (95%CI)**	***p***	***I^2***
**LM**								
Lumbar spine BMD	4	0.48(0.25, 0.71)	<0.001	88.8	4	0.66(0.55, 0.76)	<0.001	70.2
Femoral neck BMD	2	0.67(0.59, 0.75)	<0.001	76.3	3	0.58(0.38, 0.78)	<0.001	87.9
Total body BMD	7	0.52(0.32, 0.72)	<0.001	96.6	7	0.55(0.36, 0.75)	<0.001	97.1
**FM**								
Lumbar spine BMD	4	0.45(0.31, 0.58)	<0.001	71.3	4	0.23(0.04, 0.42)	0.016	76.1
Femoral neck BMD	3	0.30(0.05, 0.54)	0.018	93.1	3	0.18(0.02, 0.34)	0.029	58.9
Total body BMD	7	0.44(0.25, 0.63)	<0.001	95.3	7	0.35(0.15, 0.54)	<0.001	94.4
**BF%**								
Upper limbs BMD	2	0.30(0.12, 0.47)	0.001	32.7	2	-0.02 (-0.52, 0.49)	0.947	90.5
Lumbar spine BMD	3	0.05(-0.49, 0.59)	0.852	96.1	3	-0.35(-0.47, -0.24)	<0.001	6.8
Total body BMD	3	0.18(-0.10, 0.46)	0.199	87.2	2	-0.26(-0.65, 0.14)	0.200	90.1

Notes: LM, lean mass; FM, fat mass; BF%, body fat percentage; ES, effect size; CI, confidence interval; BMD, bone mineral density.

Supplementary Table 4. Subgroups analysis by age for correlation coefficients

	Adolescents				Children			
	**n**	**ES (95%CI)**	**p**	**I2**	**n**	**ES (95%CI)**	**p**	**I2**
**LM**								
Total body BMD	9	0.57(0.45, 0.69)	<0.001	89.6	3	0.30(-0.12, 0.73)	0.165	97.7
**FM**								
Total body BMD	9	0.43(0.27, 0.58)	<0.001	91.0	3	0.14 (-0.16, 0.44)	0.350	93.4

Notes: LM, lean mass; FM, fat mass; ES, effect size; CI, confidence interval; BMD, bone mineral density.

Supplementary Table 5. Subgroups analysis by ethnicity for regression coefficients

	White				Yellow			
	**n**	**ES (95%CI)**	***p***	***I*²**	**n**	**ES (95%CI)**	***p***	***I*²**
**LM**								
Lumbar spine BMD	8	0.44(0.16, 0.72)	0.002	99.0	6	0.57(0.42, 0.73)	<0.001	74.8
Femoral neck BMD	7	0.22(-0.05, 0.49)	0.116	90.2	6	0.66(0.50, 0.81)	<0.001	72.6
Total body BMD	14	0.53(0.34, 0.72)	<0.001	99.4	4	0.80(0.64, 0.97)	<0.001	79.0
**FM**								
Lumbar spine BMD	6	-0.01(-0.25, 0.23)	0.925	94.1	5	-0.01(-0.03, 0.02)	0.733	81.2
Femoral neck BMD	8	0.30(-0.03, 0.62)	0.071	98.8	6	-0.01(-0.01, 0.01)	0.491	73.8
Total body BMD	14	0.02(-0.05, 0.09)	0.605	96.1	9	-0.06(-0.13, 0.01)	0.072	49.7

Notes: LM, lean mass; FM, fat mass; BF%, body fat percentage; ES, effect size; CI, confidence interval; BMD, bone mineral density.

Supplementary Table 6. Subgroups analysis by sex for regression coefficients

	Female				Male			
	**n**	**ES (95%CI)**	***p***	**I^2**	**n**	**ES (95%CI)**	***p***	**I^2**
**LM**								
Lumbar spine BMD	5	0.37(0.15, 0.60)	0.001	92.8	6	0.55(0.26, 0.85)	<0.001	94.2
Femoral neck BMD	6	0.42(0.09, 0.74)	0.012	91.6	6	0.72(0.31, 1.13)	0.001	93.3
Total body BMD	7	0.51(0.25, 0.76)	<0.001	95.3	6	0.61(0.28, 0.93)	<0.001	95.5
**FM**								
Upper limbs BMD	3	0.01(-0.23, 0.25)	0.963	43.8	2	0.52(-0.34, 1.37)	0.236	84.1
Lumbar spine BMD	4	0.01(-0.01, 0.03)	0.420	68.8	5	-0.07(-0.22,0.09)	0.397	84.2
Femoral neck BMD	7	-0.01(-0.03, 0.02)	0.803	72.5	6	-0.02(-0.05, 0.02)	0.432	80.6
Total body BMD	8	0.06(-0.07, 0.19)	0.341	78.4	9	-0.03(-0.15, 0.09)	0.635	85.8
**BF%**								
Lumbar spine BMD	4	-0.15(-0.32, 0.03)	0.094	67.2	4	-0.52(-0.61, -0.43)	<0.001	90.6
Femoral neck BMD	4	-0.02(-0.09, 0.04)	0.475	83.6	4	-0.39(-0.57, -0.21)	<0.001	97.0
Total body BMD	5	-0.31(-0.64,0.03)	0.071	90.0	4	-0.19(-0.34, -0.05)	0.009	67.3

Notes: LM, lean mass; FM, fat mass; BF%, body fat percentage; ES, effect size; CI, confidence interval; BMD, bone mineral density.

Supplementary Table 7. Subgroups analysis by age for regression coefficients

	Adolescents				Children			
	**n**	**ES (95%CI)**	***p***	**I^2**	**n**	**ES (95%CI)**	***p***	**I^2**
**LM**								
Total body BMD	7	0.10 (0.07, 0.12)	<0.001	98.4	5	0.09(0.04, 0.14)	<0.001	99.2
**FM**								
Total body BMD	12	-0.02(-0.04, 0.01)	0.143	80.6	5	0.01(-0.02, 0.05)	0.496	92.7

Notes: LM, lean mass; FM, fat mass; ES, effect size; CI, confidence interval; BMD, bone mineral density.

Supplementary Table 8. Assessment of potential publication bias by Egger's test

	P	
	r	**β**
**LM**		
Upper limbs BMD	/	/
Lumbar spine BMD	0.053	0.541
Femoral neck BMD	0.056	**0.021**
Total body BMD	**<0.001**	**0.018**
**FM**		
Upper limbs BMD	/	0.053
Lumbar spine BMD	**0.030**	0.449
Femoral neck BMD	0.952	0.352
Total body BMD	**0.001**	0.542
**BF%**		
Upper limbs BMD	0.083	/
Lumbar spine BMD	0.737	**0.044**
Femoral neck BMD	0.390	0.286
Total body BMD	0.740	**0.025**

Notes: BMD, bone mineral density; bold, $p < 0.050$.

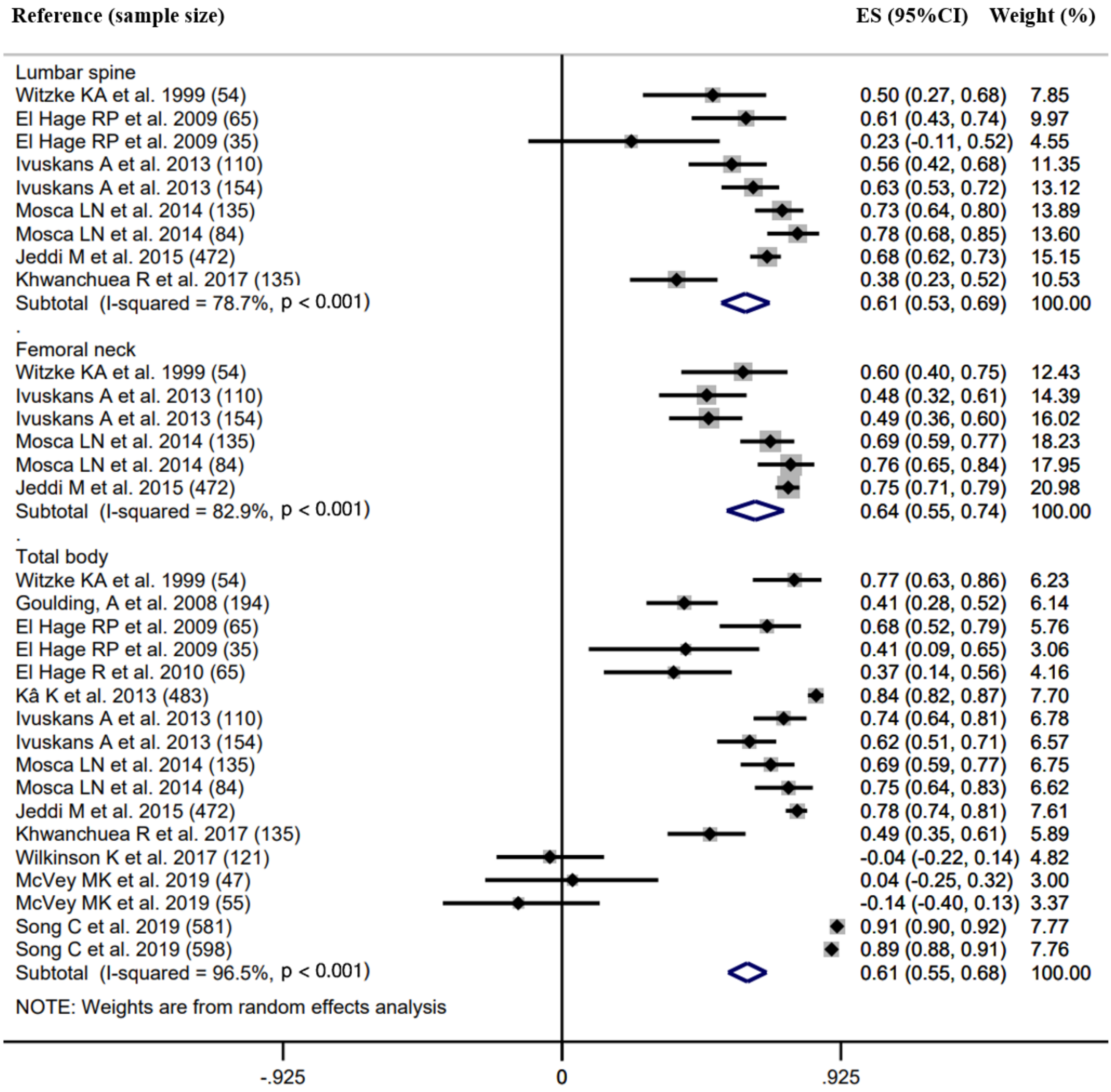

Supplementary Figure 1. Forest plot of correlation coefficient between lean mass and bone mineral density at lumber spine, femoral neck and total body, respectively. The effect size (ES) and 95% confidence interval (CI) for fully adjusted random effects are depicted for each study.

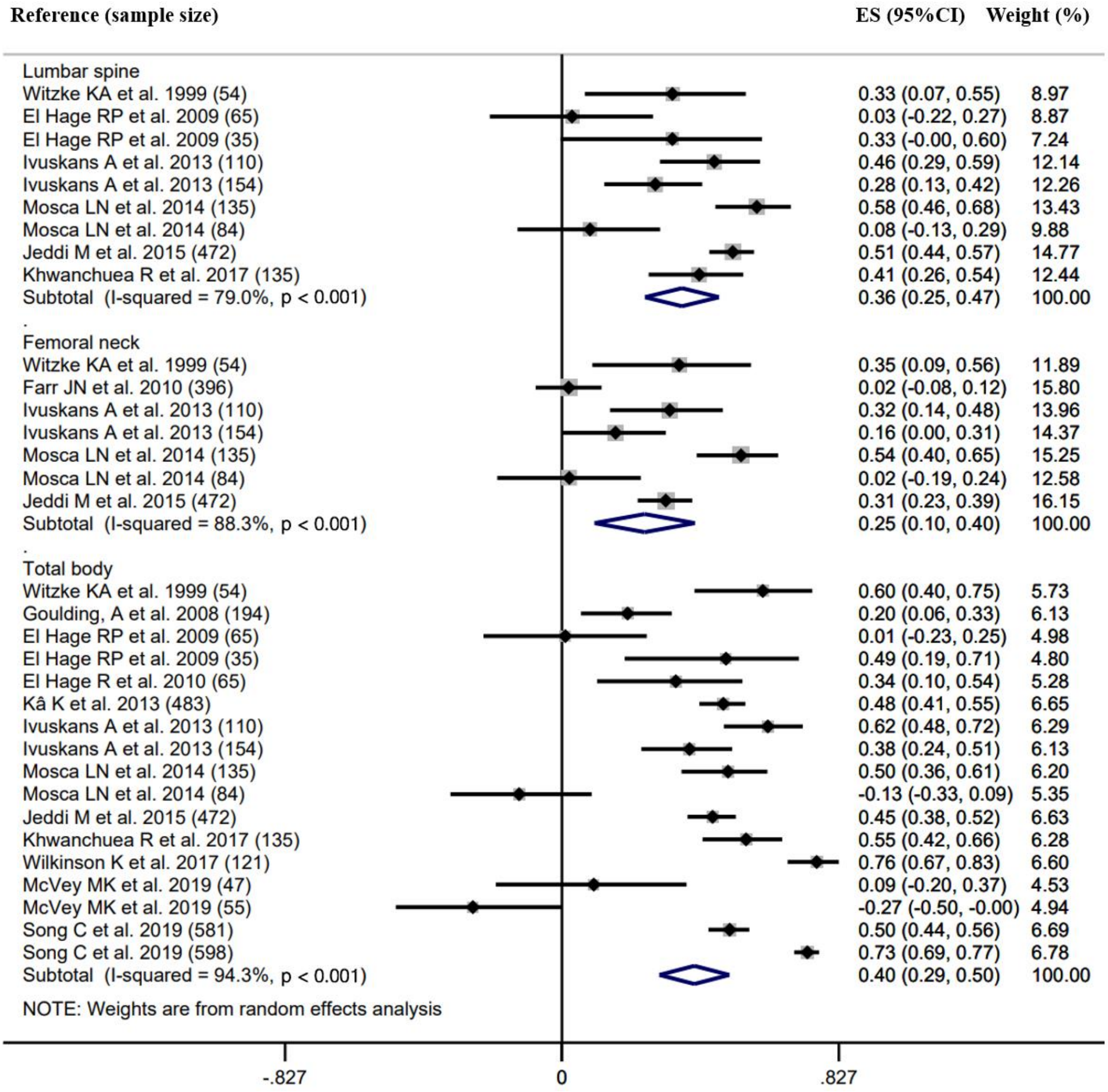

Supplementary Figure 2. Forest plot of correlation coefficient between fat mass and bone mineral density at lumber spine, femoral neck and total body, respectively. The effect size (ES) and 95% confidence interval (CI) for fully adjusted random effects are depicted for each study.

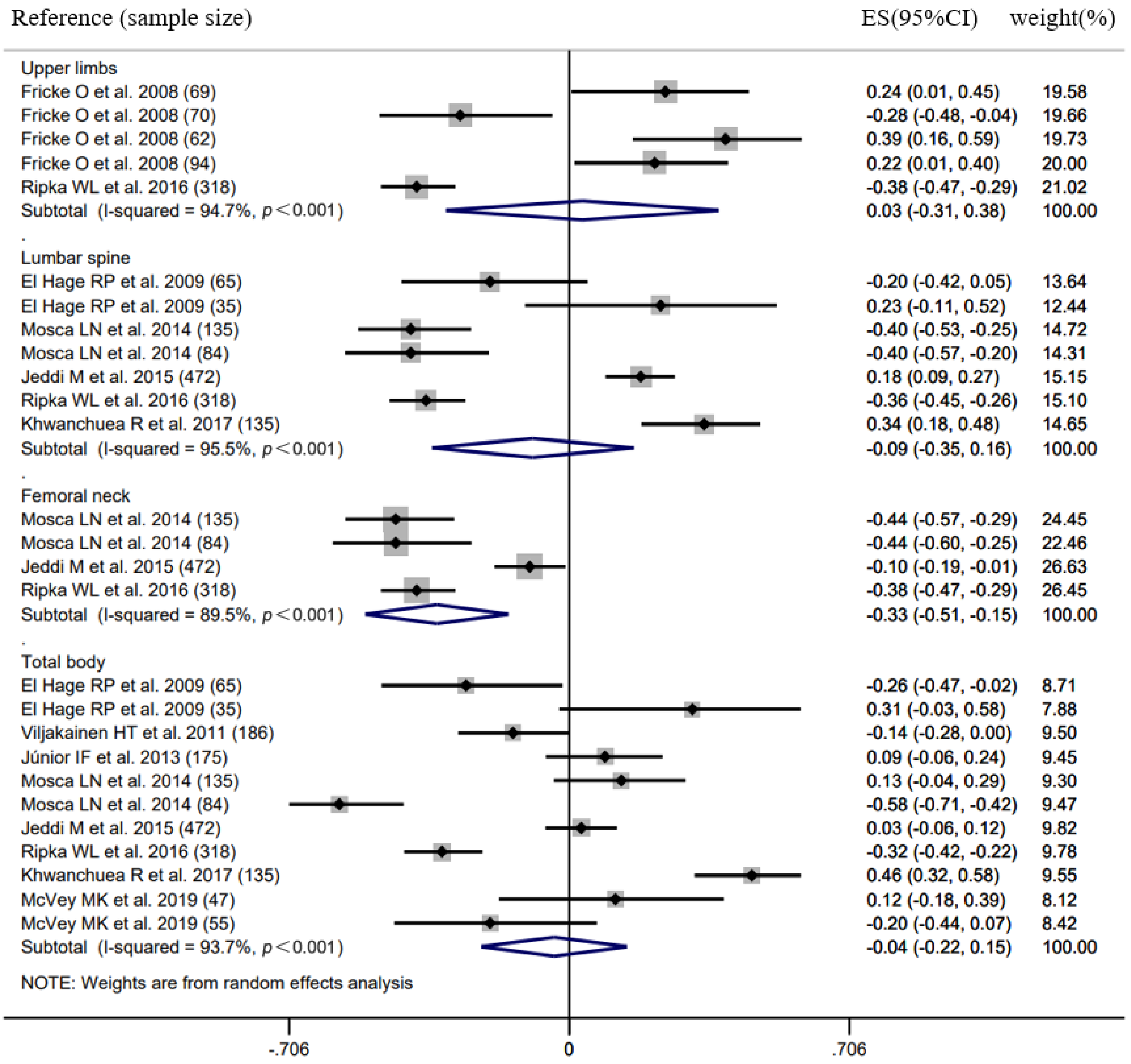

Supplementary Figure 3. Forest plot of correlation coefficient between body fat percentage and bone mineral density at upper limbs, lumber spine, femoral neck and total body, respectively. The effect size (ES) and 95% confidence interval (CI) for fully adjusted random effects are depicted for each study.

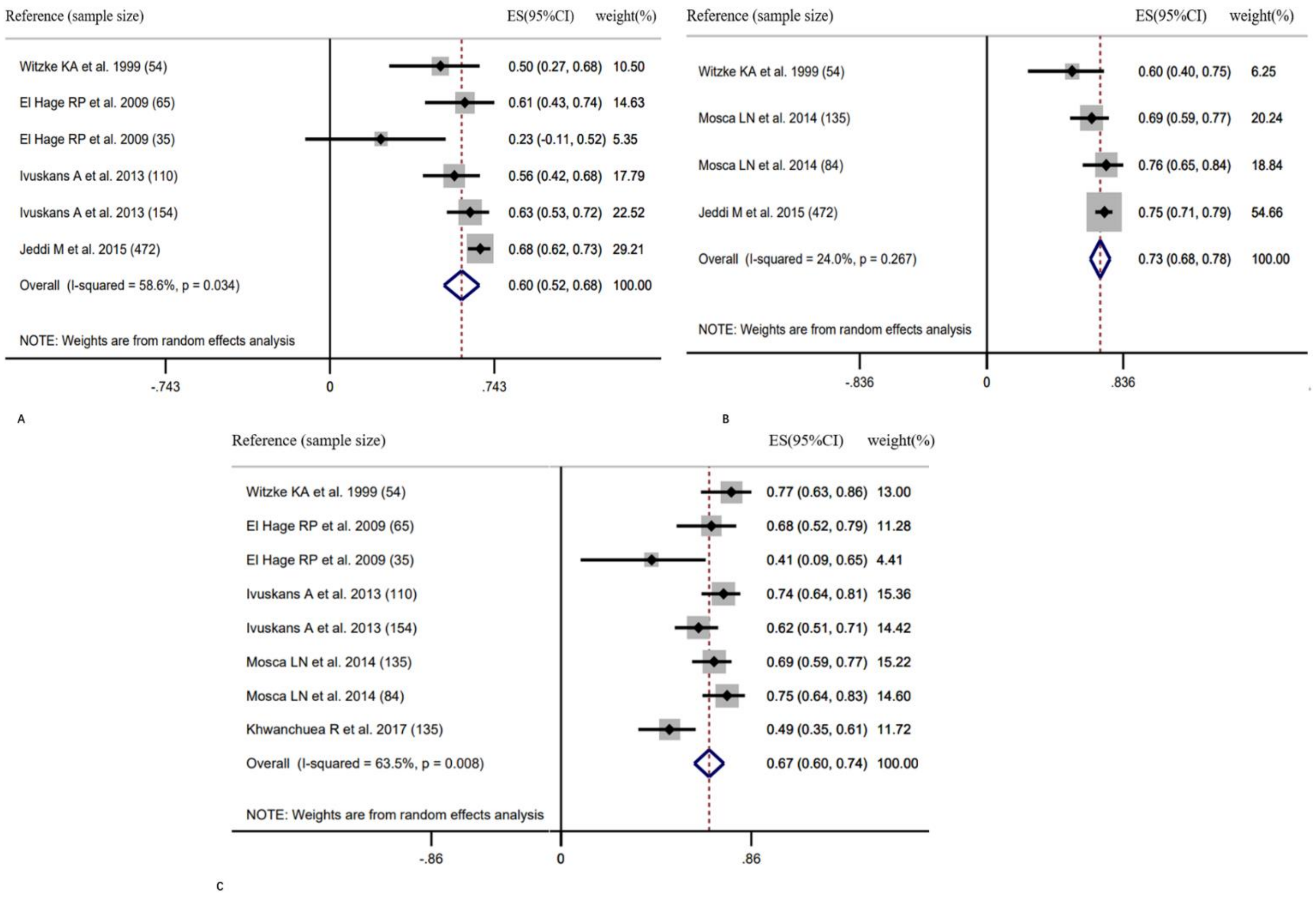

Supplementary Figure 4. Forest plot of the correlation coefficient of lean mass and bone mineral density at lumbar spine (A), femoral neck (B) and total body (C) after the exclusion of studies with large heterogeneity.

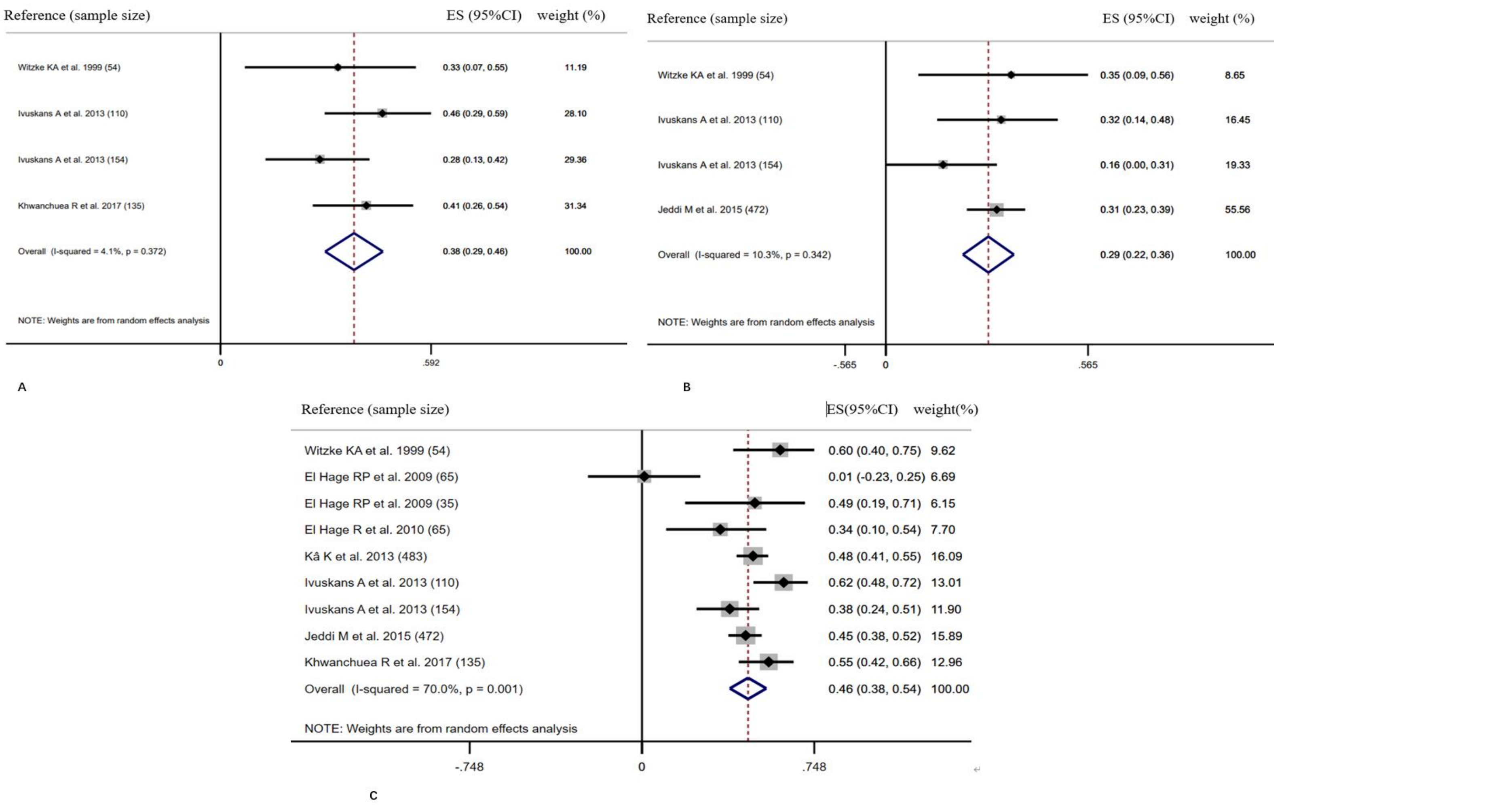

Supplementary Figure 5. Forest plot of the correlation coefficient of fat mass and bone mineral density at lumbar spine (A), femoral neck (B) and total body (C) after the exclusion of studies with large heterogeneity.

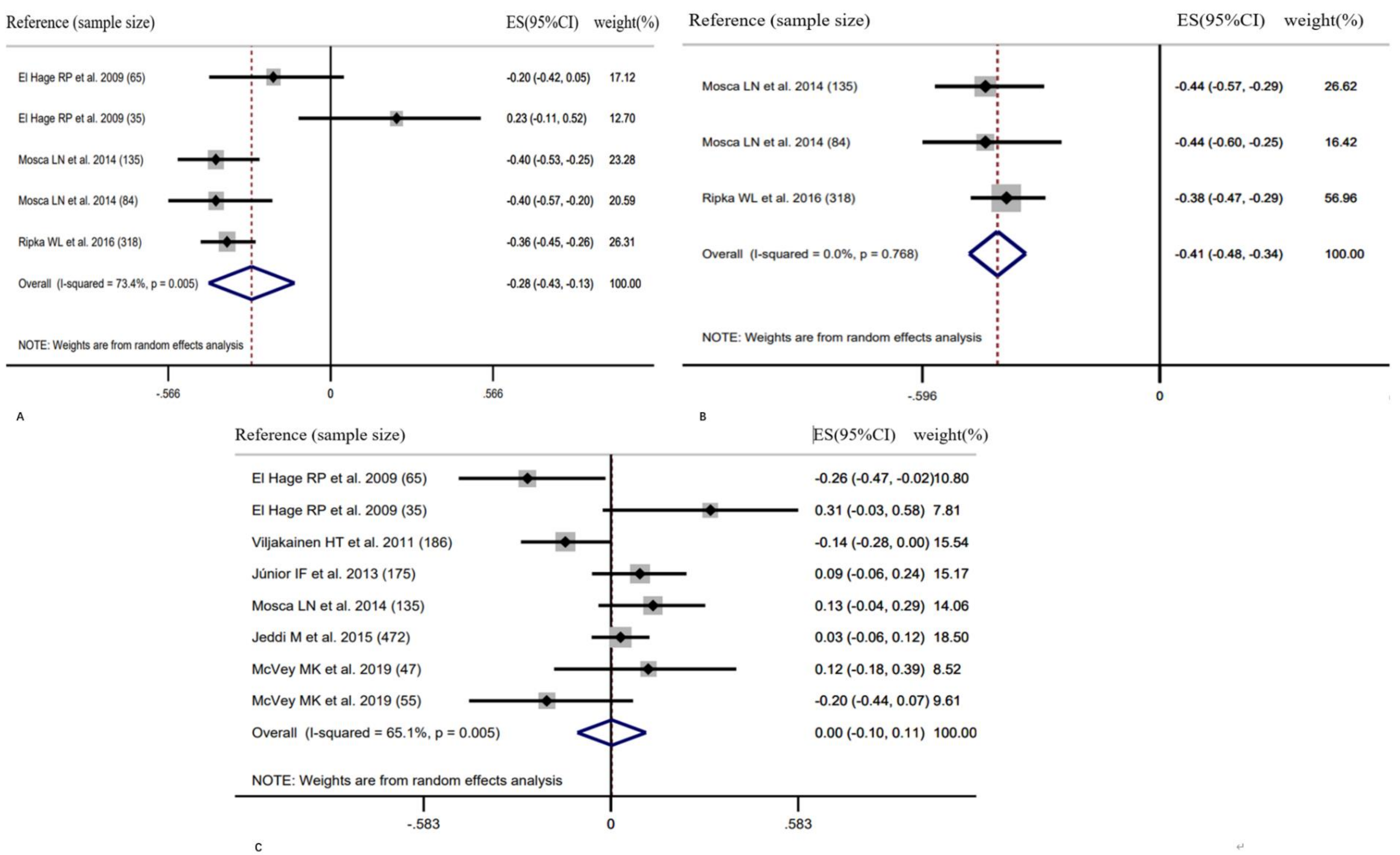

Supplementary Figure 6. Forest plot of the correlation coefficient of body fat percentage and bone mineral density at lumbar spine (A), femoral neck (B) and total body (C) after the exclusion of studies with large heterogeneity.

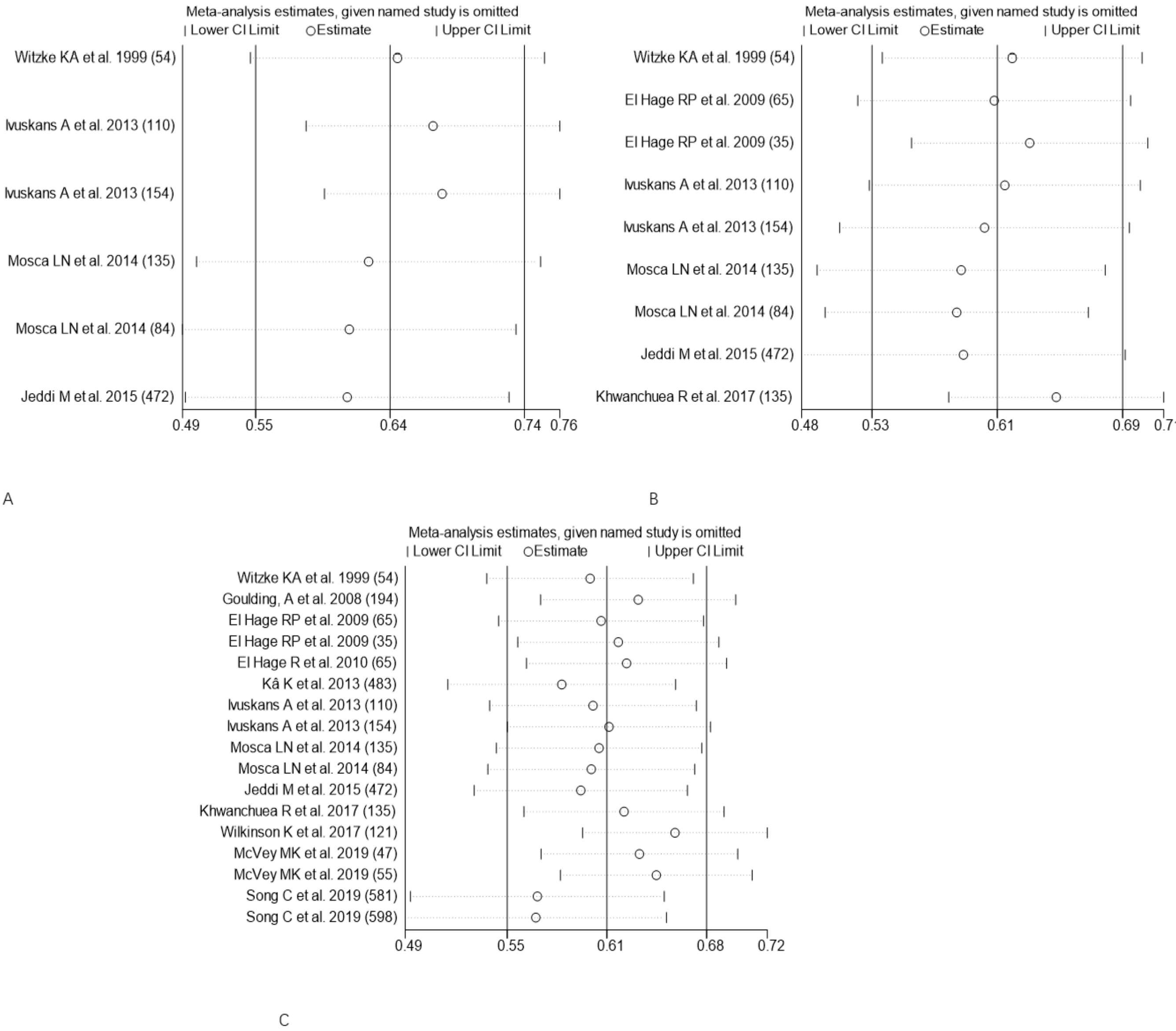

Supplementary Figure 7. Plots of sensitivity analyses by removing 1 study at a time (A. Four studies reported the correlation coefficient of lean mass and femoral neck bone mineral density (BMD); B. Six studies reported the correlation coefficient of lean mass and lumbar spine BMD; C. Twelve studies reported the correlation coefficient of lean mass and total body BMD).

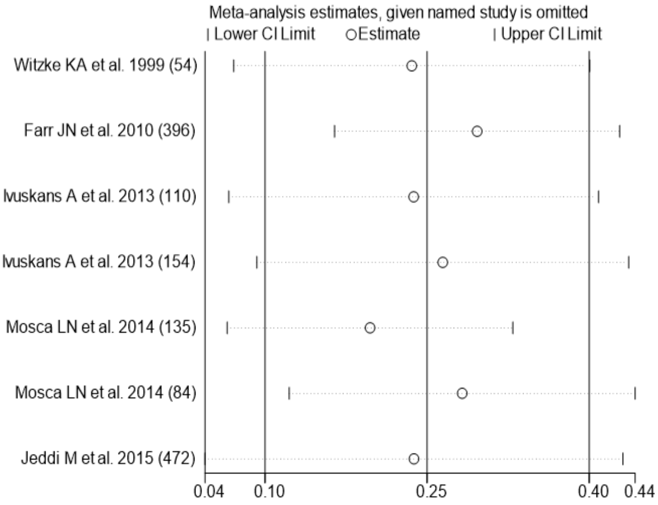

A

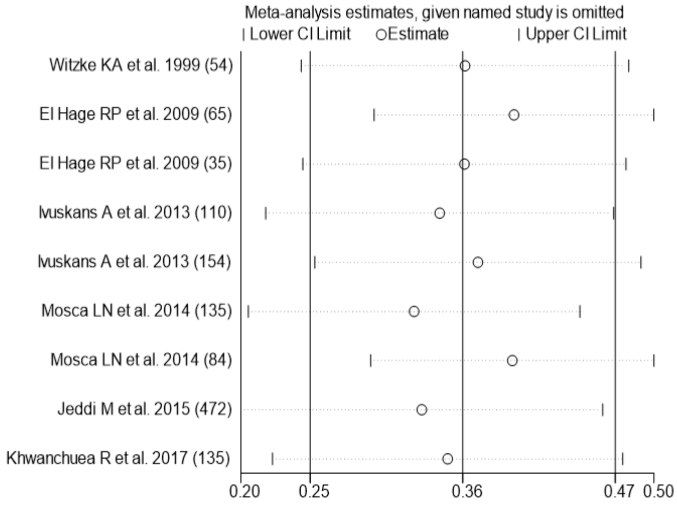

B

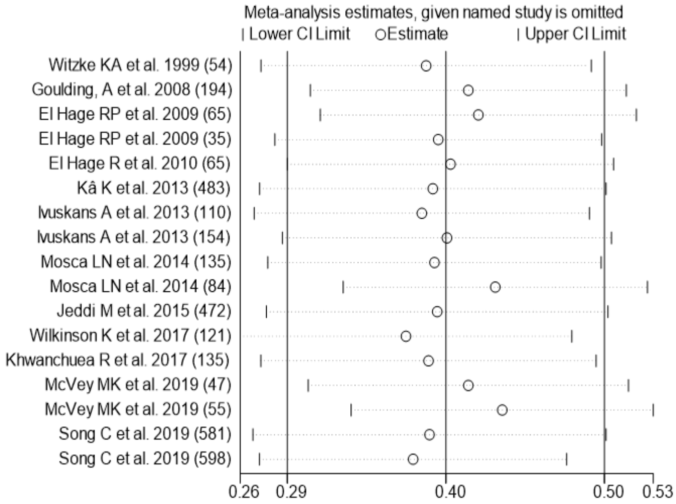

C

Supplementary Figure 8. Plots of sensitivity analyses by removing 1 study at a time (A. Five studies reported the correlation coefficient of fat mass and femoral neck BMD; B. Six studies reported the correlation coefficient of fat mass and lumbar spine BMD; C. Twelve studies reported the correlation coefficient of fat mass and total body BMD.)

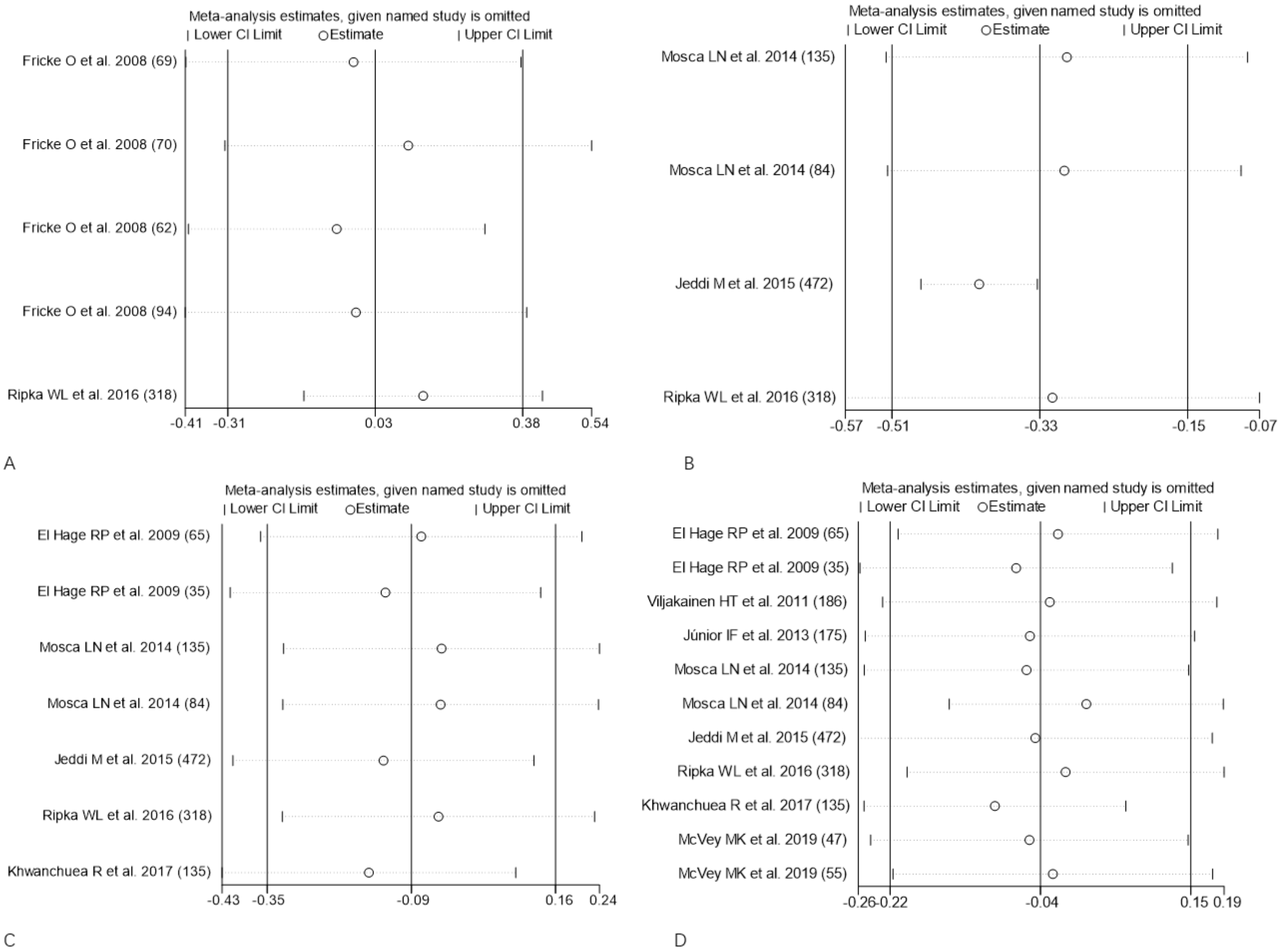

Supplementary Figure 9. Plots of sensitivity analyses by removing 1 study at a time (A. Two studies reported the correlation coefficient of body fat percentage and upper limbs bone mineral density (BMD); B. Three studies reported the correlation coefficient of body fat percentage and femoral neck BMD; C. Five studies reported the correlation coefficient of body fat percentage and lumbar spine BMD; D. Eight studies reported the correlation coefficient of body fat percentage and total body BMD.)

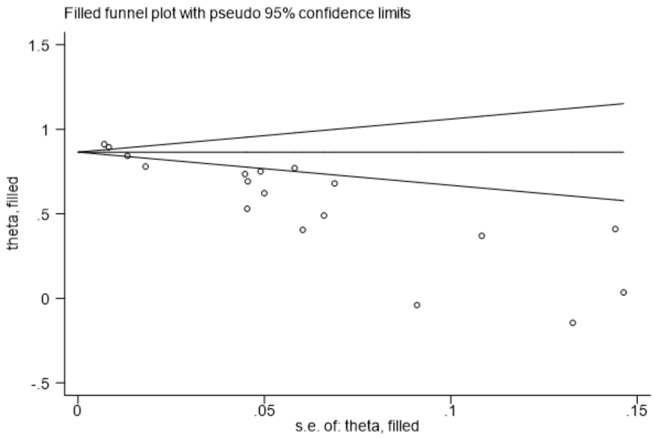

A

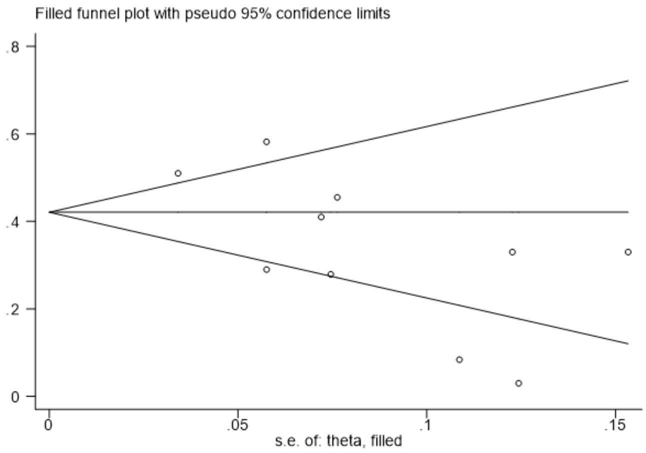

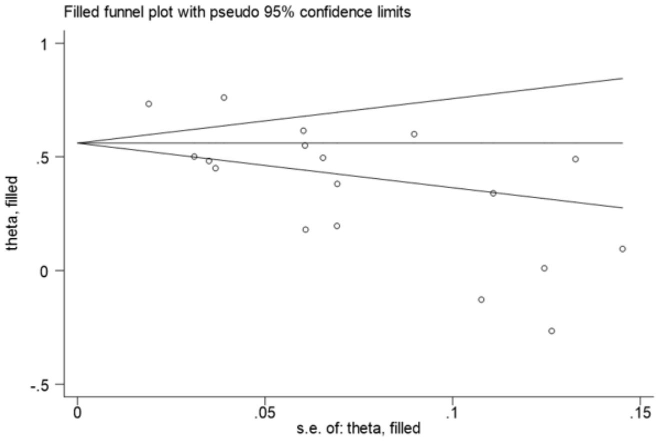

B C

Supplementary Figure 10. Plots of Duval and Tweedie's trim and fill test (A. No studies trimmed and filled for the correlation coefficient of lean mass and total body bone mineral density (BMD); B. No studies trimmed and filled for the correlation coefficient of fat mass and lumbar spine BMD; C. No studies trimmed and filled for the correlation coefficient of fat mass and total body

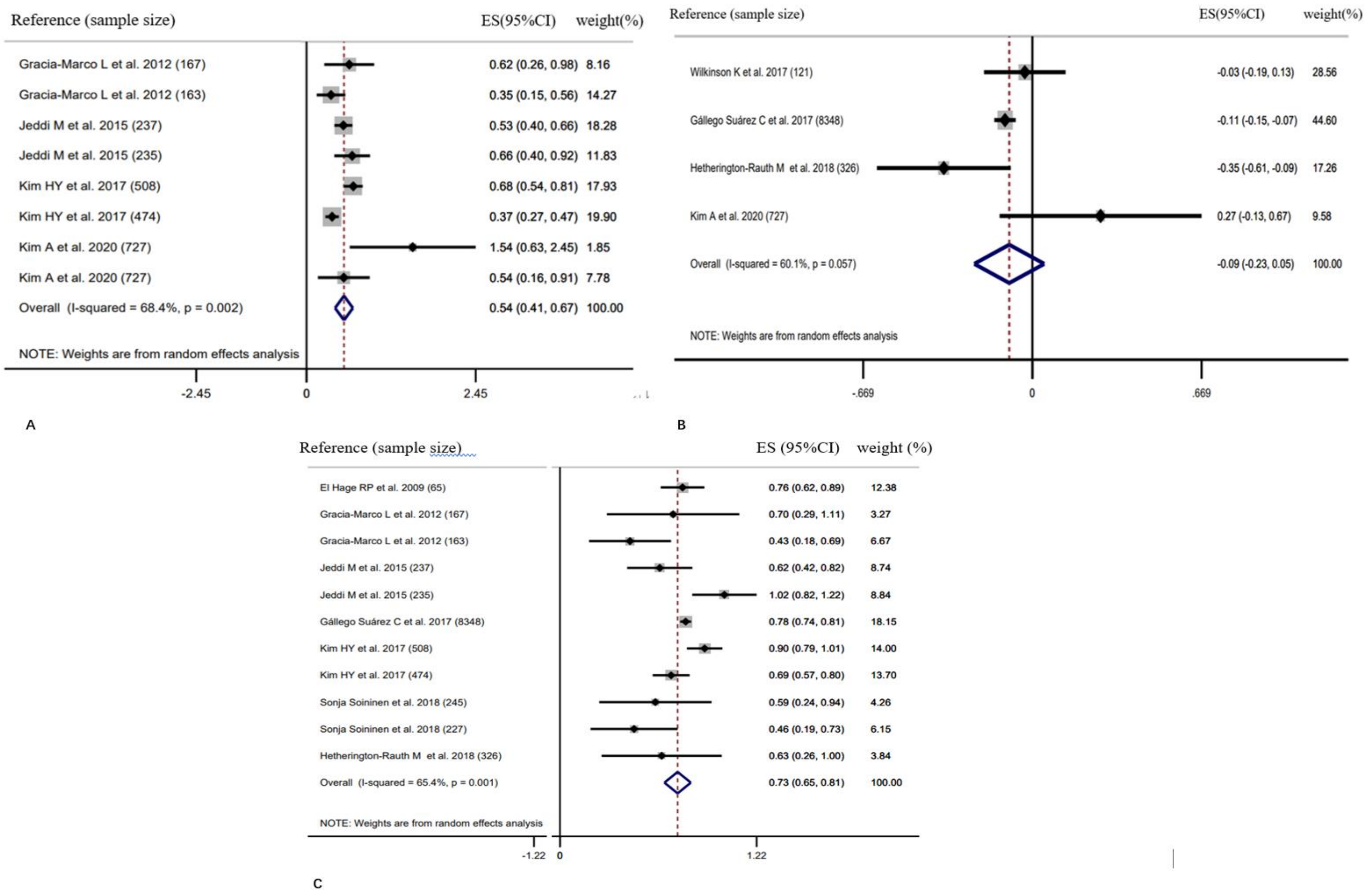

Supplementary Figure 11. Forest plot of the regression coefficient of lean mass and bone mineral density at lumbar spine (A), femoral neck (B) and total body (C) after exclusion of study that has large heterogeneity.

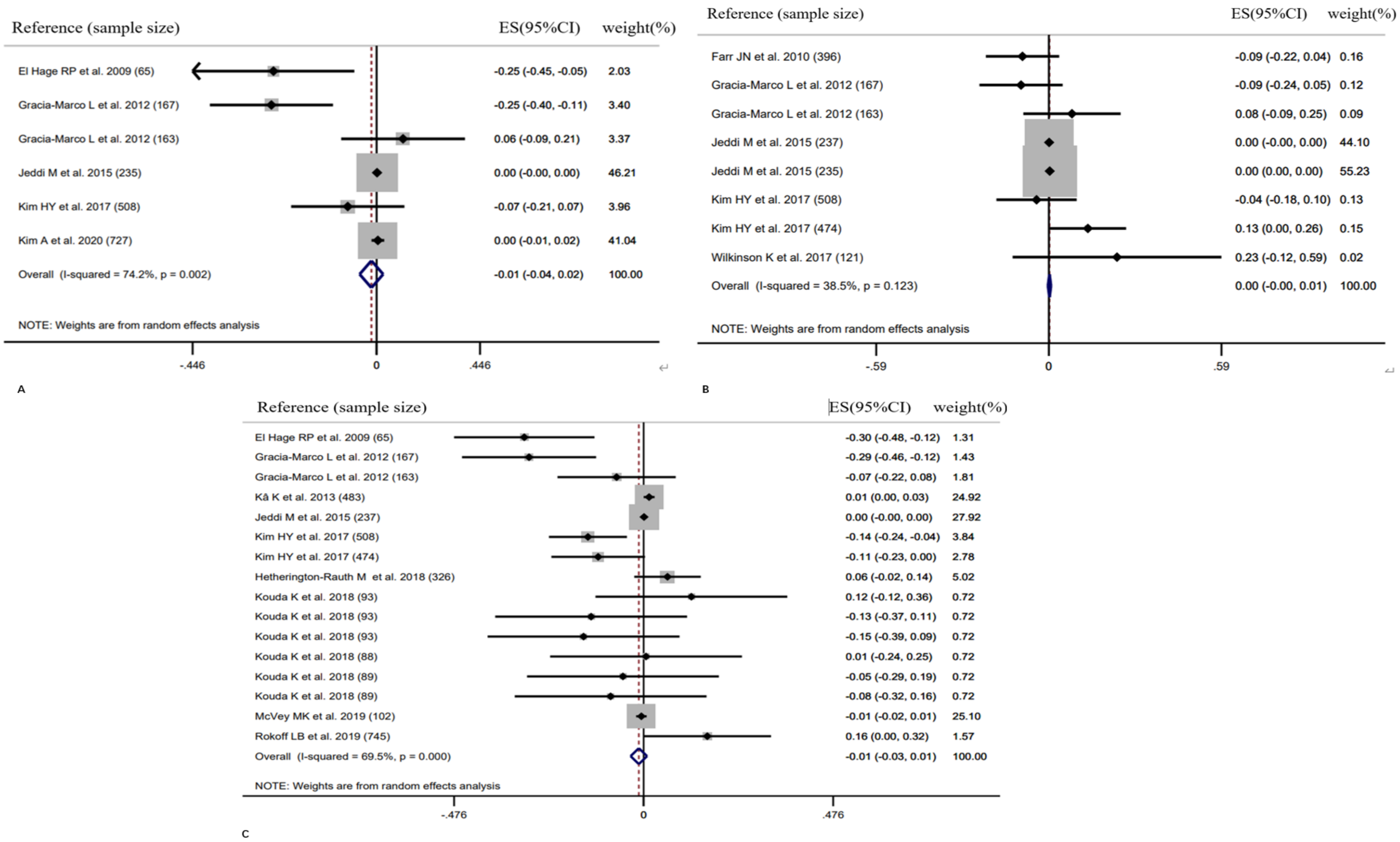

Supplementary Figure 12. Forest plot of the regression coefficient of fat mass and bone mineral density at lumbar spine (A), femoral neck (B) and total body (C) after the exclusion of studies with large heterogeneity.

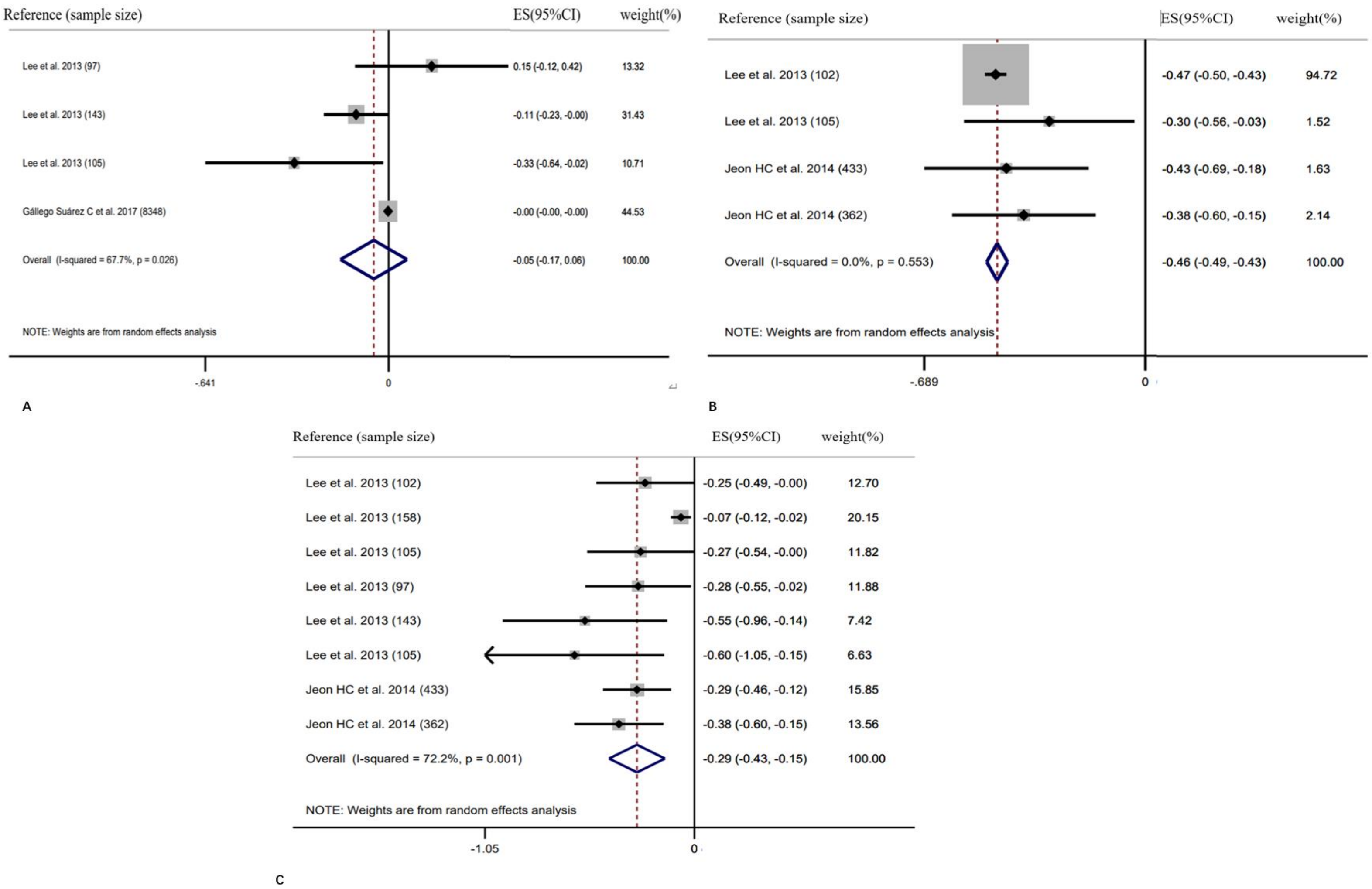

Supplementary Figure 13. Forest plot of the regression coefficient of body fat percentage and bone mineral density at lumbar spine (A), femoral neck (B) and total body (C) after the exclusion of studies with large heterogeneity.

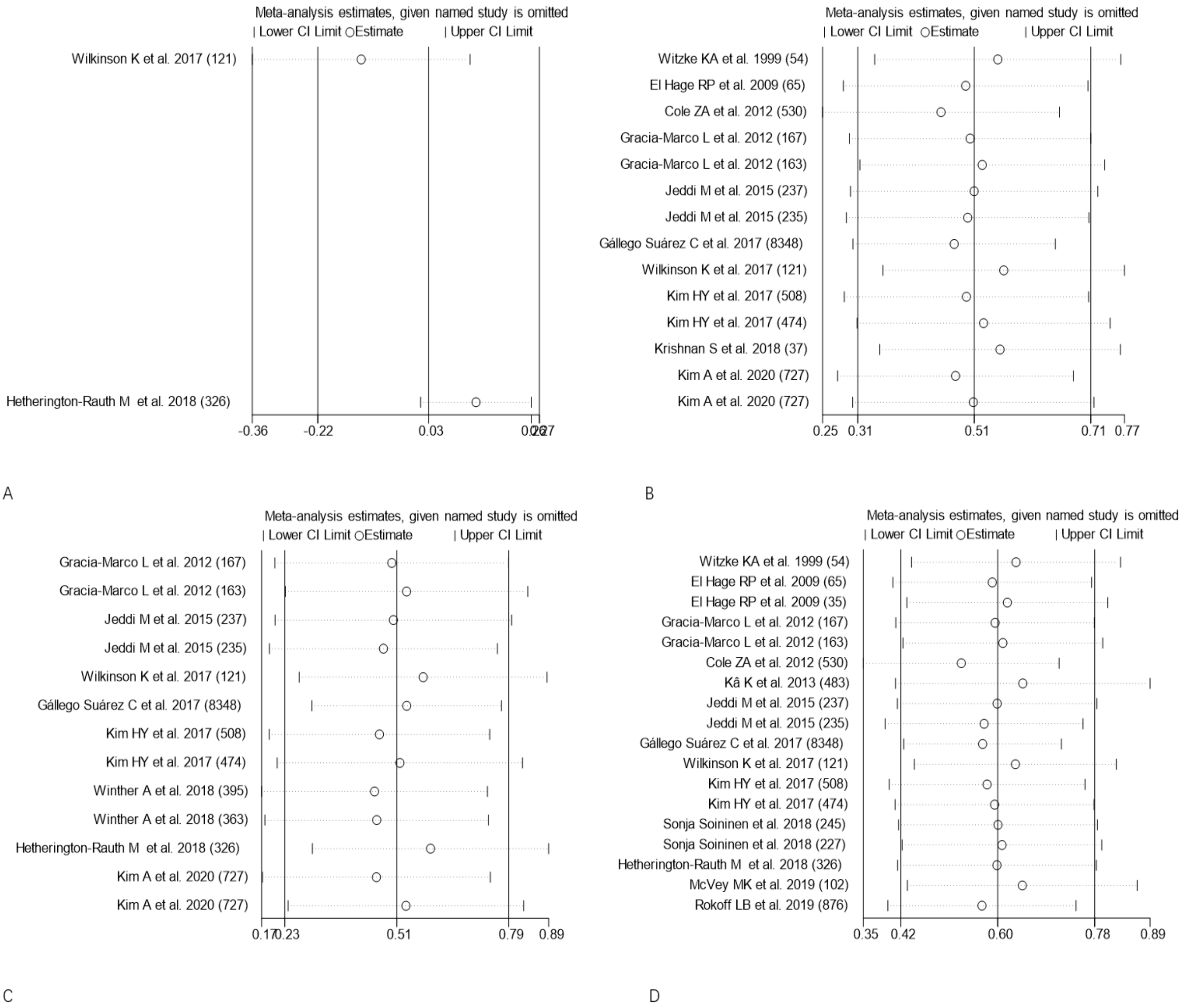

Supplementary Figure 14. Plots of sensitivity analyses by removing 1 study at a time (A. Two studies reported the regression coefficient of lean mass and upper limbs bone mineral density (BMD); B. Ten studies reported the regression coefficient of lean mass and lumbar spine BMD; C. Eight studies reported the regression coefficient of lean mass and femoral neck BMD; D. Thirteen studies reported the regression coefficient of lean mass and total body BMD.)

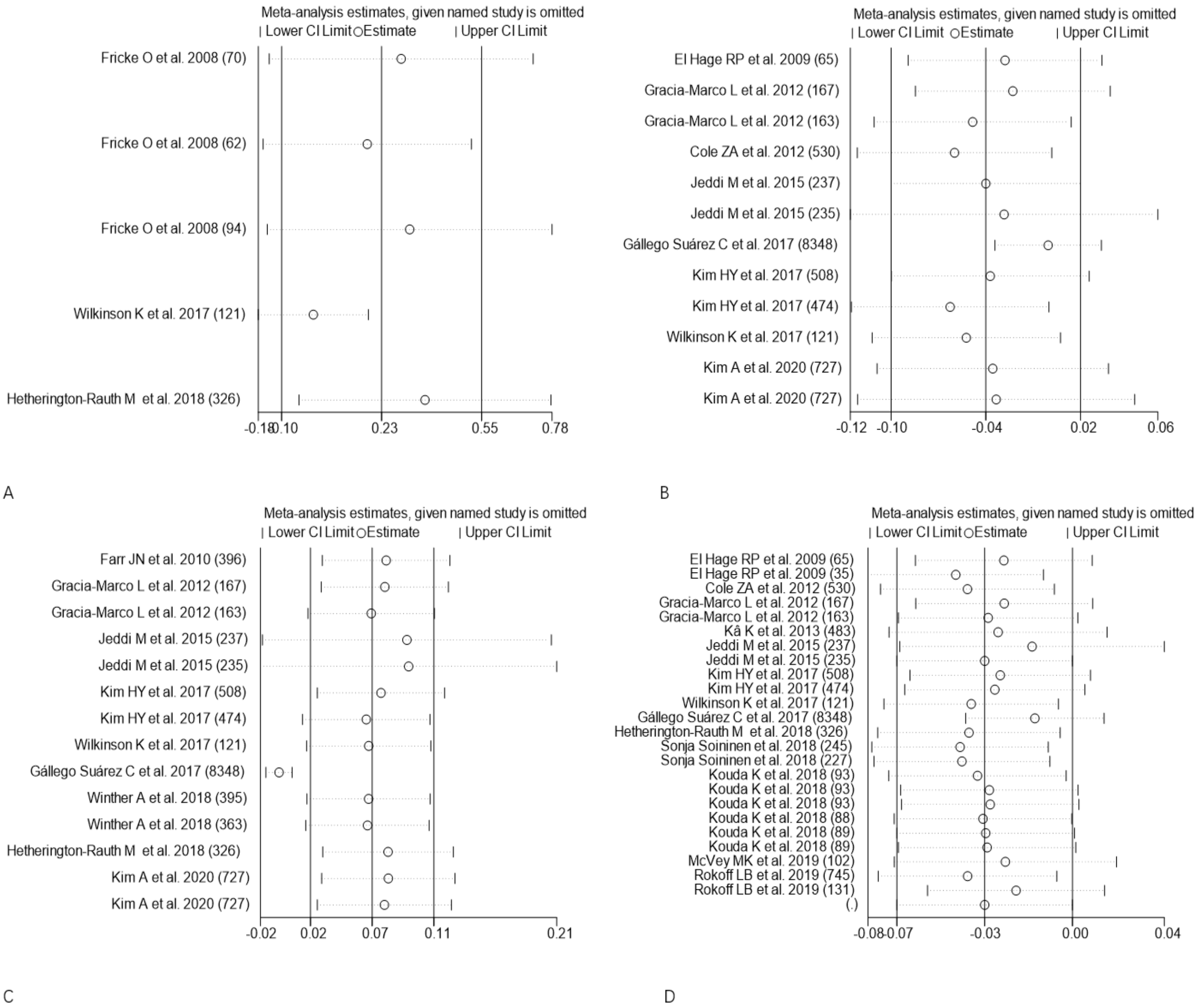

Supplementary Figure 15. Plots of sensitivity analyses by removing 1 study at a time (A. Three studies reported the regression coefficient of fat mass and upper limbs bone mineral density (BMD); B. Eight studies reported the regression coefficient of fat mass and lumbar spine BMD; C. Nine studies reported the regression coefficient of fat mass and femoral neck BMD; D. Thirteen studies reported the regression coefficient of fat mass and total body BMD.)

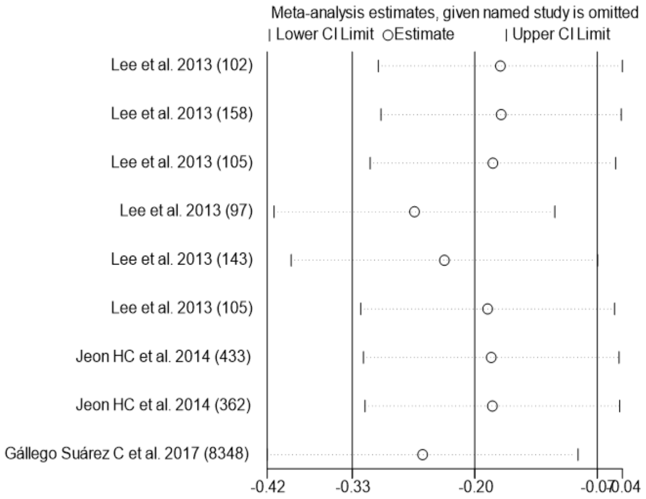

A

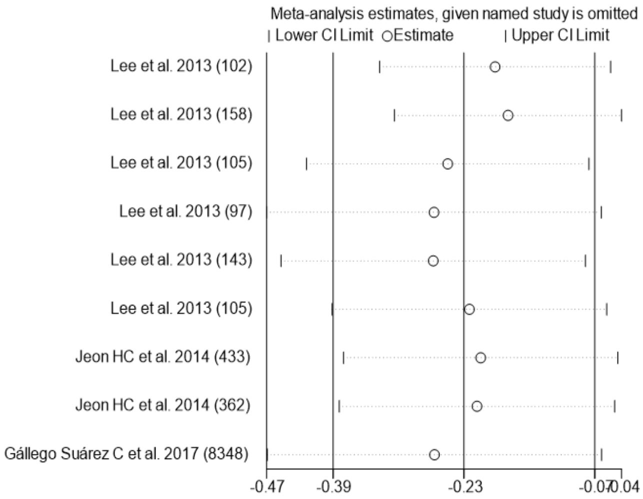

B

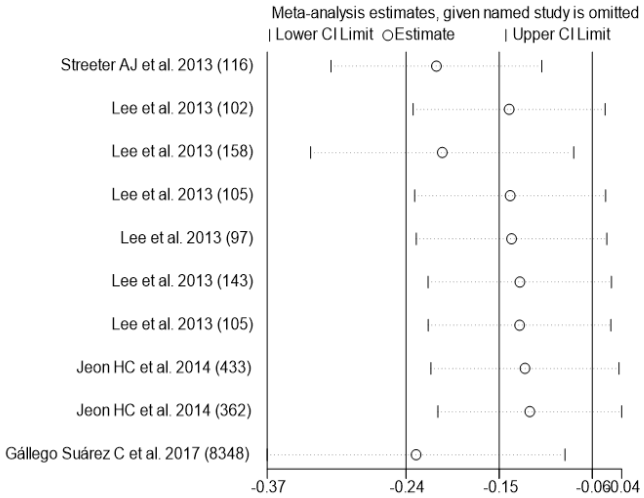

C

Supplementary Figure 16. Plots of sensitivity analyses by removing 1 study at a time (A. Three studies reported the regression coefficient of body fat percentage and lumbar spine bone mineral density (BMD); B. Three studies reported the regression coefficient of body fat percentage and femoral neck BMD; C. Four studies reported the regression coefficient of body fat percentage and total body BMD.)

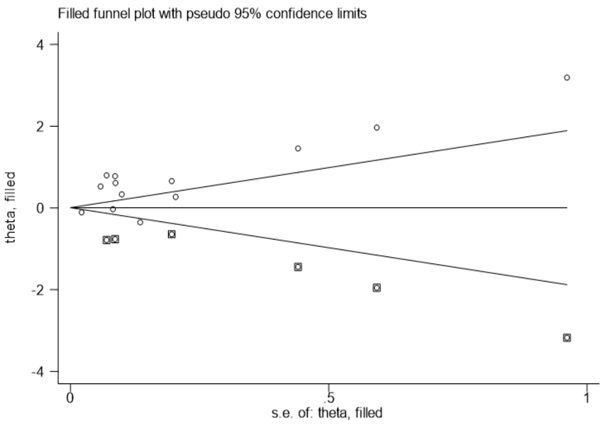

A

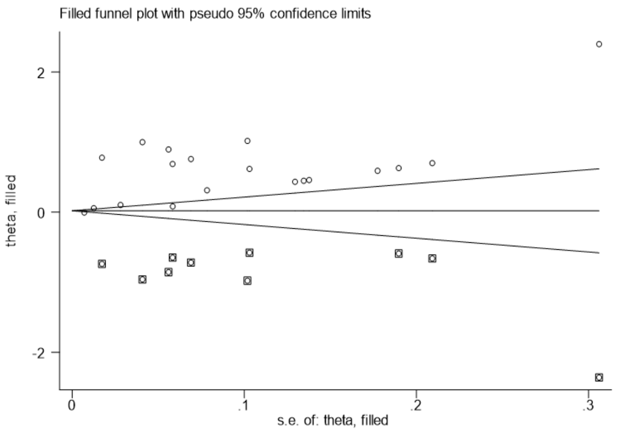

B

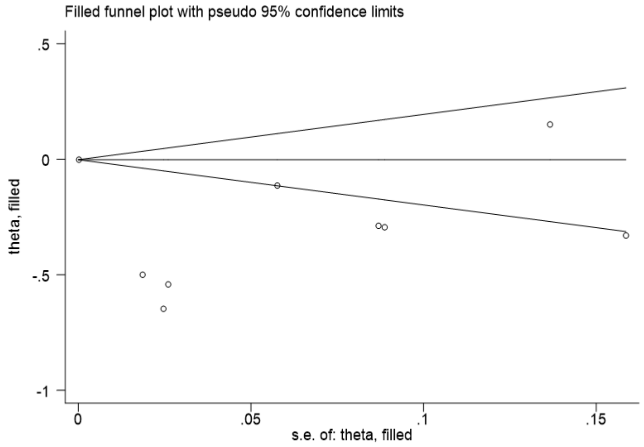

C

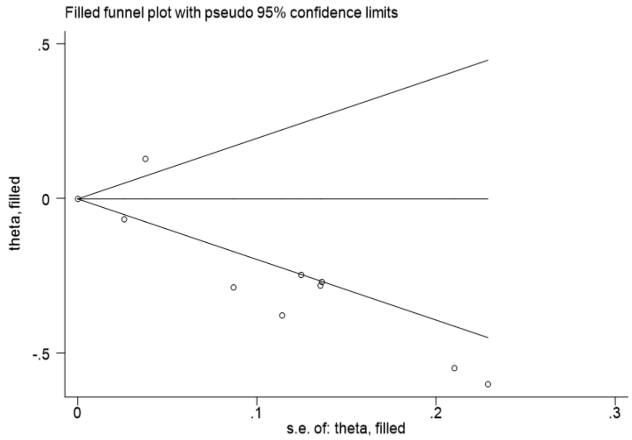

D

Supplementary Figure 17. Plots of Duval and Tweedie's trim and fill test (A. Six items trimmed and filled for the regression coefficient of lean mass and femoral neck bone mineral density (BMD); B. Ten items trimmed and filled for the regression coefficient of lean mass and total body BMD; C. No studies trimmed and filled for the regression coefficient of body fat percentage and lumbar spine BMD; D. No studies trimmed and filled for the regression coefficient of body fat percentage and total body BMD).
